# A continuous-time stochastic Boolean model provides a quantitative description of the budding yeast cell cycle

**DOI:** 10.1038/s41598-022-24302-6

**Published:** 2022-11-24

**Authors:** Teeraphan Laomettachit, Pavel Kraikivski, John J. Tyson

**Affiliations:** 1 Bioinformatics and Systems Biology Program, School of Bioresources and Technology, King Mongkut’s University of Technology Thonburi, Bangkok, 10150 Thailand; 2 Theoretical and Computational Physics Group, Center of Excellence in Theoretical and Computational Science, King Mongkut’s University of Technology Thonburi, Bangkok, 10140 Thailand; 3 Division of Systems Biology, Academy of Integrated Science, Virginia Polytechnic Institute and State University, Blacksburg, VA 24061 USA; 4 Department of Biological Sciences, Virginia Polytechnic Institute and State University, Blacksburg, VA 24061 USA

**Keywords:** Cell biology, Computational biology and bioinformatics, Molecular biology, Systems biology

## Abstract

The cell division cycle is regulated by a complex network of interacting genes and proteins. The control system has been modeled in many ways, from qualitative Boolean switching-networks to quantitative differential equations and highly detailed stochastic simulations. Here we develop a continuous-time stochastic model using seven Boolean variables to represent the activities of major regulators of the budding yeast cell cycle plus one continuous variable representing cell growth. The Boolean variables are updated asynchronously by logical rules based on known biochemistry of the cell-cycle control system using Gillespie’s stochastic simulation algorithm. Time and cell size are updated continuously. By simulating a population of yeast cells, we calculate statistical properties of cell cycle progression that can be compared directly to experimental measurements. Perturbations of the normal sequence of events indicate that the cell cycle is 91% robust to random ‘flips’ of the Boolean variables, but 9% of the perturbations induce lethal mistakes in cell cycle progression. This simple, hybrid Boolean model gives a good account of the growth and division of budding yeast cells, suggesting that this modeling approach may be as accurate as detailed reaction-kinetic modeling with considerably less demands on estimating rate constants.

## Introduction

Budding yeast, *Saccharomyces cerevisiae*, is a model organism for studying regulation of the eukaryotic cell cycle. The complex gene-protein interaction network controlling the yeast cell division cycle has been modeled by several different mathematical techniques, including discrete Boolean networks and nonlinear ordinary differential equations (ODE). ODE models track variables with continuous values (concentrations or activities of proteins) in continuous time. Boolean models, on the other hand, assign variables to discrete states (0 or 1) in discrete time (0, 1, 2, 3, …).

Chen et al.^1,2^ developed a highly detailed ODE model to describe the synthesis, degradation, and modification of proteins in the cell-cycle pathway. The model captured essential features of the budding yeast cell-cycle control system observed in wild-type and > 100 mutants. Later models incorporated probabilistic components to simulate the stochastic nature of cell-cycle progression^3,4^. These models, when properly parametrized, match experimental results with exquisite quantitative detail, but the models employ a large number of kinetic parameters that must be measured from experiments or estimated with care.

At the other extreme, Li et al.^5^ proposed a Boolean model tracking the discrete states (0 = off or 1 = on) of eleven proteins that are major regulators in the budding yeast cell cycle. The rules for updating the Boolean variables, $B_{i} \left( {t + 1} \right) = F_{i} \left( {B_{1} \left( t \right),B_{2} \left( t \right), \ldots ,B_{11} \left( t \right)} \right),i = 1, \ldots 11$, were derived from known properties of the molecular regulatory network, and the variables were updated synchronously, i.e., if two or more variables are predicted to change state in the next time interval, then they all change state simultaneously. Simulations of the model revealed that, of the 2^11^ = 2048 initial states, 1764 progress to a single state of the eleven proteins corresponding to a ‘stationary G_1_’ phase of the cell cycle. (The remaining 284 initial states end up in one of six other fixed points.) Furthermore, the large basin of attraction drains quickly onto a sequence of thirteen states (see Fig. 2 and Table 2 of Li et al.^5^) corresponding to the known sequence of molecular transitions as a budding yeast cell progresses from late G_1_ to S phase, mitosis and cell division into stationary (early) G_1_ phase. Li et al. interpreted their model as evidence that the yeast cell-cycle network, from a qualitative point-of-view, is ‘robustly designed.’ However, this conclusion is dependent on updating the Boolean model synchronously. If the model is updated asynchronously (i.e., if several variables are predicted to change state, then only one of them—chosen randomly or by some predetermined rule—actually changes state), then the thirteen-state sequence (the ‘cell-cycle highway’) is no longer a robust attractor of the dynamics, as shown in Supplementary Text S1. Nonetheless, a few simple changes of the Boolean rules restore robustness to the model, even in the case of asynchronous updating (Supplementary Text S1). Evidently, the robustness of Boolean models is sensitively dependent on the rules and on the updating scheme. Furthermore, as the authors point out, the number of time steps in each cell-cycle phase does not reflect actual duration, because a ‘time step’ in a discrete Boolean model is an arbitrary ‘tick’ of the metronome that coordinates updating of the Boolean variables. For this reason, Boolean simulations cannot be compared quantitatively to experimental measurements of cell cycle durations, which is a major drawback of a Boolean framework.

Since the pioneering study of Li et al., many other authors have proposed Boolean models of cell cycle progression in budding yeast^6–9^, fission yeast^10,11^, and mammalian cells^12–15^ (Supplementary Table S1). Their models vary in complexity from ‘standard’ Boolean (with synchronous, asynchronous or mixed updating)^5–7,10–12^ to ‘hybrid’ models (Boolean + ODEs)^13,14^ to ‘stochastic’ models (random asynchronous updating)^15^ to models with subcellular compartments^9^ and a ‘comprehensive’ model tracking 357 mRNAs and proteins participating 1238 elemental states (post-translational modifications and complex formation)^8^. Our intention is not to improve on these studies but to consider the consequences of random asynchronous updating on a Boolean model of the budding yeast cell cycle. We update the Boolean variables asynchronously and stochastically in continuous time, using the ‘Boolean Kinetic Monte-Carlo’ (BKMC) approach developed by Stoll et al.^15^ As an example of their approach, Stoll et al. applied BKMC to the mammalian cell cycle model of Faure et al.^12^ but did not explore the model in any detail or compare simulations to experimental data.

In this paper, we apply BKMC to a modified version of the Li et al. model. First, we reduced the model to seven discrete variables by lumping together some proteins that perform analogous functions in Li’s network, in order to focus on the consequences of asynchronous updating and stochastic progression. Second, we introduced a continuous variable for cell size, because growth of yeast cells to a ‘critical size’ is known to play a crucial role in progression through the cell cycle^16–32^.

Using this model, we perform thousands of simulations (‘realizations’) of a population of budding yeast cells undergoing unsynchronized growth and division. From this collection we calculate statistical properties of cell-cycle progression that can be compared directly to experimental observations. The Boolean rules are crafted to produce a repetitive sequence of transitions (a cell-cycle highway) that corresponds to the normal progression of molecular events observed in the budding yeast cell cycle. Our model includes the effects of cell growth on cell cycle progression, so we can compute the distributions of cell size at birth and division and the correlations of cell size with cell-cycle phase durations. By comparing model simulations to experimental observations, we show that the BKMC approach (random asynchronous updating of a Boolean model) can account in quantitative detail for many characteristics of cell cycle progression in wild-type budding yeast cells. Finally, we consider all single-state ‘flips’ of the Boolean variables away from the cell-cycle highway, in order to address the robustness of the model to ‘small’ perturbations of the control system. About 9% these flips induce lethal mistakes, in circumstances where lethality would be expected from the underlying molecular biology of the control system.

## Methods

### The model

In all eukaryotic cells, events of the cell division cycle are coordinated by cyclin-dependent protein kinases, whose activities initiate DNA synthesis (S-phase cyclins) and mitosis (M-phase cyclins). As cells exit mitosis, these cyclins are ubiquitinated and degraded by ubiquitin ligase enzymes and proteasomes, respectively. In budding yeast, the S-phase cyclins are Clb5 and Clb6, the M-phase cyclins are Clb1 and Clb2, and another pair of cyclins (Cln1 and Cln2) initiate bud emergence. Our model employs seven Boolean variables to track the activities of these key regulators of the budding yeast cell cycle. The Boolean variables, which can be either 0 (off, low activity) or 1 (on, high activity), are:

- Cdh1 = combined activities of Cdh1 and Sic1 (respectively, the ubiquitin ligase and stoichiometric inhibitor that oppose Clb5/6 and Clb1/2 activities in G_1_ phase);
- SBF = combined activities of SBF and MBF (the transcription factors for Cln1/2 and Clb5/6);
- Cln2 = combined activities of Cln1 and Cln2;
- Clb5 = combined activities of Clb5 and Clb6;
- Clb2_G_ = combined activities of Clb1 and Clb2 in prophase;
- Clb2_M_ = higher activities of Clb1 and Clb2 in metaphase;
- Cdc20 = combined activities of Cdc20 and Cdc14 (respectively, the ubiquitin ligase and protein phosphatase that oppose Clb5/6 and Clb1/2 activities as cells exit mitosis).

The ‘state’ of the dynamical system is given by a septuplet of Boolean variables: (Cdh1, SBF, Cln2, Clb5, Clb2_G_, Clb2_M_, Cdc20); for example, a cell in early G_1_ phase would be in state (1000000). The Boolean variables are updated asynchronously, i.e., only a single variable changes its activity (from 0 to 1 or 1 to 0) in each time step.

Our model also includes a variable representing the ‘size’ of a budding yeast cell, in order to account for the considerable body of evidence for size-control of the yeast cell cycle^16–32^. Although cell size is most often measured as volume (fL), ‘geometric size’ is presumably a proxy for some still-unknown ‘physiological size.’ Despite lingering uncertainties about how yeast cells measure their ‘size,’ we introduce a continuous variable, called ‘Size(*t*),’ which increases exponentially in time with a ‘mass doubling time’ of approx. 100 min (appropriate for well-nourished yeast cells in a laboratory culture) and is apportioned unequally between mother and daughter cells at division.

We update the variables in each simulation step by a Boolean version of Gillespie’s stochastic simulation algorithm^33^. The general framework is:

1. The possible new activity of each variable is determined by a logical function
  1 $$B_{i}^{*} = F_{i} \left( {B_{1} \left( t \right),B_{2} \left( t \right), \ldots ,B_{7} \left( t \right)} \right),$$
  where $F_{i} \left( \cdots \right)$ takes the current activities of all variables, *B*_*j*_, to determine the potentially new activity of variable $i\left( {B_{i}^{*} } \right)$ in the next iteration.
2. We calculate the propensity that each variable will change its activity
  2 $$P_{i} = p_{i} \cdot \left| {B_{i}^{*} - B_{i} \left( t \right)} \right|,$$
  where *p*_i_ is a constant (the probability per unit time the *B*_*i*_ will make a potential change).
3. Of all the variables with a potential to change, only one is selected with a probability proportional to its propensity calculated in step 2. Technically, let
  3 $$P_{0} = \mathop \sum \limits_{i = 1}^{7} P_{i}$$
  be the sum of all propensities. The index of the selected activity change is the smallest integer *j* that satisfies $\mathop \sum \limits_{i = 1}^{j} P_{i} > r_{1} \cdot P_{0}$ where *r*_1_ is a random number drawn from a uniform distribution between 0 and 1.
4. The time interval at which the activity change chosen in step 3 occurs is calculated as
  4 $$\Delta t = - \log \left( {r_{2} } \right)/P_{0} ,$$
  where *r*_2_ is another random number drawn from a uniform distribution between 0 and 1, independently of *r*_1_.
5. Once the index *j* is selected, and $\Delta t$ is determined, we set
  5 $$B_{j} \left( {t + \Delta t} \right) = B_{j}^{*} ,\;{\text{and}}\;B_{i \ne j} \left( {t + \Delta t} \right) = B_{i} \left( t \right).$$
6. Lastly, we update Size using an exponential growth function ^34^
  6 $${\text{Size}}\left( {t + \Delta t} \right) = {\text{Size}}\left( t \right) \cdot e^{\mu \cdot \Delta t} ,$$
  where $\mu$ is the specific growth rate of the cell.

The steps 1–6 are repeated iteratively until the total time of the simulation reaches a specified value.

### The Boolean updating rules

**Figure 1:**
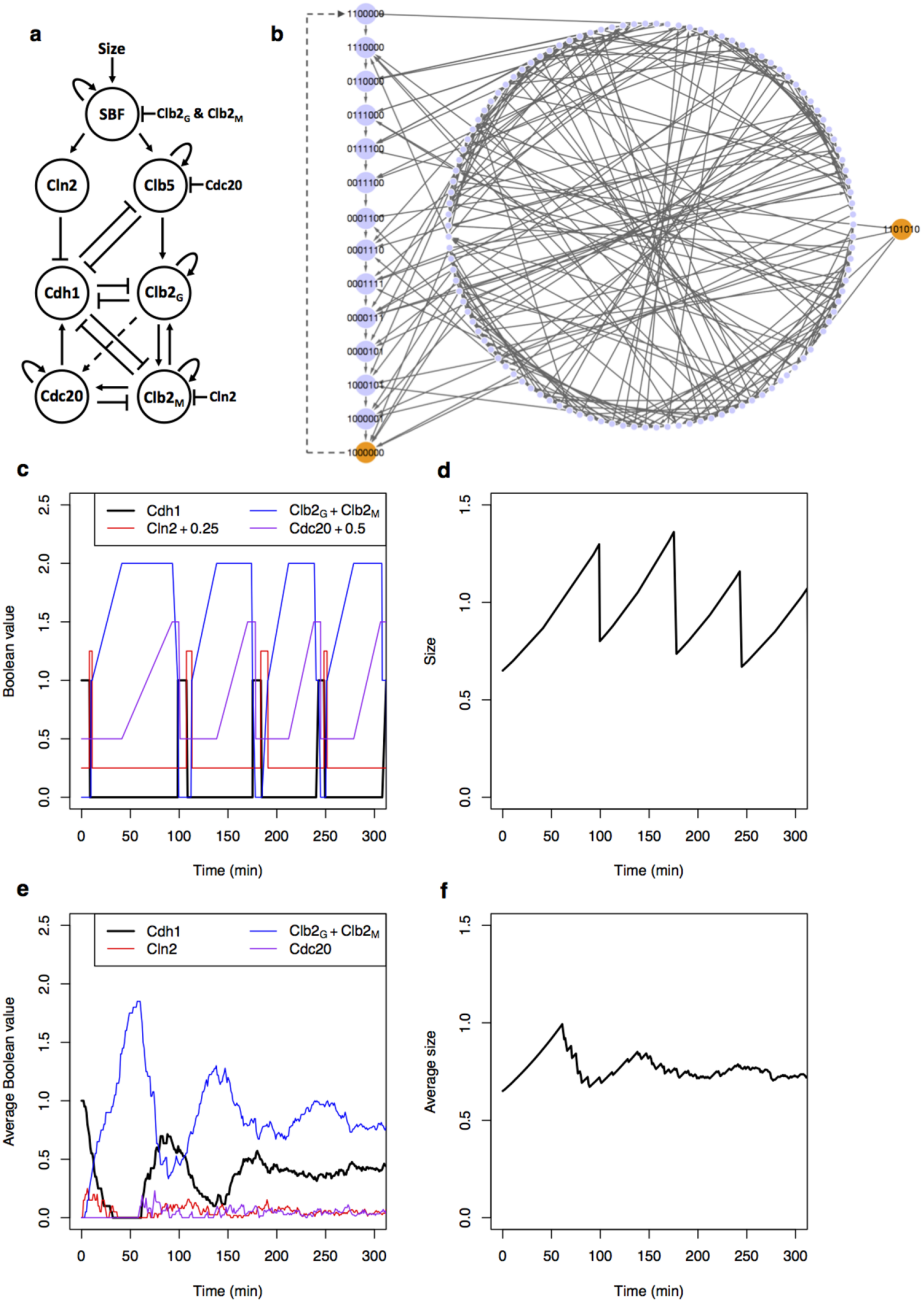
A hybrid Boolean model of the budding yeast cell cycle. (**a**) Simplified diagram of the molecular interactions governing progression through the budding yeast cell division cycle. Arrows, dashed arrows, and blunt-ended lines represent activation, stabilization, and inhibition, respectively. See Eqs. (7–13) and the main text for a full description of the interactions. (**b**) State transitions of the Boolean dynamics. Each node represents one of the 128 protein states on seven Boolean variables. Arrows point from the current state to the next possible states. In the absence of cell growth, the network is a directed acyclic graph (as all nodes can be topologically sorted using Kahn’s algorithm^56^) with one root node (1101010) and one sink node (1000000) (orange nodes). The 14 nodes to the left, labeled with 7-digit numbers, represent the globally attracting ‘cell-cycle highway’ (see Table 2) from ‘late G_1_’ (1100000) through S, G_2_, M, CD to the stable ‘early G_1_’ state (1000000). The dashed arrow from 1000000 to 1100000 is the Start transition (the activation of SBF and MBF), made possible by an increase in cell size above a critical threshold *S*_0_. Starting from any state on the big circular component, the cell-cycle highway is reached—on average—in three steps (for the full distribution of transition lengths, see Supplementary Fig. S1). (**c** and **d**) Time evolution of Boolean variables and cell size, respectively, following a lineage of mother cells through 3^+^ cycles. Activities of Cln2 and Cdc20 in panel c are offset by 0.25 and 0.5, respectively, for clearer visualization. (**e** and **f**) Values of the same variables, averaged over a population of initially size-selected cells. A population is started from 20 cells of Size = 0.65 (the average size of a mother cell after division), which are followed along with their progeny (both mother and daughter cells) for 400 min. Averages are calculated over all cells extant at time *t*.

The seven Boolean variables regulate one another according to logical functions that are derived from known interactions of the budding yeast cell-cycle control system (Fig. 1a), as follows.

Cdh1 is phosphorylated and inactivated by Cln- and Clb-dependent kinases (Cln2, Clb5, Clb2_G_, or Clb2_M_ in our model)^35^. During mitotic exit, Cdh1 is dephosphorylated and activated by Cdc14 (which is included as part of the ‘Cdc20’ variable in our model)^36^. We assume that Cdc20 (i.e., Cdc14) activates Cdh1 even in the presence of Clb2_G_. These logical relations are implemented by Eq. (7):

7 $${\text{Cdh1}}^{*} = \sim\! \left. {\left( {\left. {{\text{Cln2}}} \right|\left. {{\text{Clb5}}} \right|\left. {{\text{Clb2}}_{{\text{G}}} } \right|{\text{Clb2}}_{{\text{M}}} } \right)} \right|{\left( {{\text{Cdc2}}0\& \!\sim\! \left( {\left. {{\text{Cln2}}} \right|\left. {{\text{Clb5}}} \right|{\text{Clb2}}_{{\text{M}}} } \right)} \right)}.$$

The logical relations are ‘AND’ &, ‘OR’ |, and ‘NOT’ ~ .

The transcription factor SBF (i.e., SBF and MBF) is activated when the cell grows to a sufficiently large size^17^. We assume SBF can be activated only if Size is greater than a critical size (*S*_0_) and then with a probability proportional to (Size − *S*_0_)^2^. The critical size for a cell is assigned randomly at birth from a lognormal(meanlog, sdlog) distribution with meanlog = log(*S*_0_mean_) and sdlog = *S*_0_CV_, where *S*_0_mean_ and *S*_0_CV_ are (approximately) the mean and coefficient of variation of size at birth. Once turned on, SBF maintains its own activity until it is inactivated by Clb2 (Clb2_G_ or Clb2_M_)^37^. These logical relations are implemented by Eq. (8):

8 $$\begin{aligned} {\text{SBF}}^{*} = & \left. {\left( {\left( {{\text{ state}} = {1}000000 \, } \right)\& \left( {{\text{ Size }} > S_{0} } \right)\& \left( {r_{0} < \, \left( {{\text{Size }} - S_{0} } \right)^{{2}} } \right)} \right)} \right| \\ & \left( {{\text{ SBF}}\& \!\sim\! \left( {\left. {{\text{Clb2}}_{{\text{G}}} } \right|{\text{Clb2}}_{{\text{M}}} } \right)} \right), \\ \end{aligned}$$

where state 1000000 represents a newborn cell with active Cdh1 and the other variables inactive, and *r*_0_ is a random number drawn from a uniform distribution between 0 and 1.

SBF promotes the production of Cln2 and Clb5^38–40^. Cln2 is an unstable protein, so its activity drops rapidly after SBF is turned off. On the other hand, Clb5 maintains its production via a positive feedback loop^39^, and Clb5 degradation is promoted by Cdh1 or Cdc20^41^. These relations are implemented by Eqs. (9) and (10):

9 $${\text{Cln2}}^{*} = {\text{SBF}}$$

10 $${\text{Clb5}}^{*} = \left( {\left. {{\text{Clb5}}} \right|{\text{SBF}}} \right)\& \!\sim\! \left( {\left. {{\text{Cdh1}}} \right|{\text{Cdc2}}0} \right)$$

Clb5 enhances the production of Clb2 (Clb2_G_ and Clb2_M_)^42^. Clb2 also promotes its own production by activating its transcription factor Mcm1^43^, which is not included in the model. A high level of Clb2 (Clb2_M_) can be degraded by both Cdh1 and Cdc20^44^, but a residual level of Clb2 (Clb2_G_) can only be degraded by Cdh1^44^. In addition, we assume that Cln2 suppresses the activation of Clb2_M_ for reasons to be explained shortly. These relations are implemented in Eqs. (11) and (12)

11 $${\text{Clb2}}_{{\text{G}}}^{*} = \left. {{\text{Clb2}}_{{\text{M}}} } \right|\left( {\left( {\left. {{\text{Clb5}}} \right|{\text{Clb2}}_{{\text{G}}} } \right)\& \!\sim\! {\text{Cdh1}}} \right)$$

12 $${\text{Clb2}}_{{\text{M}}}^{*} = \left( {\left. {{\text{Clb2}}_{{\text{G}}} } \right|{\text{Clb2}}_{{\text{M}}} } \right)\& \!\sim\! {\text{Cdh1}}\& \left( {\left. {\!\sim\! {\text{Cdc2}}0} \right|{\text{Clb5}}} \right)\& \!\sim\! {\text{Cln2}}$$

Cdc20 synthesis is stimulated by Clb2^45^. In our model, we assume that Cdc20 is activated by the high level of Clb2 (Clb2_M_). Once Cdc20 is activated, its activity can be maintained by the activity of a residual level of Clb2 (Clb2_G_), as provided in Eq. (13):

13 $${\text{Cdc2}}0^{*} = \left. {{\text{Clb2}}_{{\text{M}}} } \right|\left( {{\text{Clb2}}_{{\text{G}}} \& {\text{Cdc2}}0 \, } \right)$$

The propensity that each variable changes its activity is given by Eqs. (14–20)

14 $$P_{{1}} = \left| {{\text{ Cdh1}}^*{-}{\text{Cdh1}}\left( t \right)} \right| \cdot p_{{{\text{Cdh1}}}}$$

15 $$P_{{2}} = \left| {{\text{ SBF}}^*{-}{\text{SBF}}\left( t \right)} \right| \cdot p_{{{\text{SBF}}}}$$

16 $$P_{{3}} = \left| {{\text{ Cln2}}^*{-}{\text{Cln2}}\left( t \right)} \right| \cdot p_{{{\text{Cln2}}}}$$

17 $$P_{{4}} = \left| {{\text{Clb5}}^*{-}{\text{Clb5}}\left( {\text{t}} \right)} \right| \cdot p_{{{\text{Clb5}}}}$$

18 $$P_{{5}} = \left| {{\text{Clb2}}_{{\text{G}}} ^*{-}{\text{Clb2}}_{{\text{G}}} \left( t \right)} \right| \cdot p_{{{\text{Clb2G}}}}$$

19 $$P_{{6}} = \left| {{\text{Clb2}}_{{\text{M}}} ^*{-}{\text{Clb2}}_{{\text{M}}} \left( t \right)} \right| \cdot p_{{{\text{Clb2M}}}}$$

20 $$P_{{7}} = \left| {{\text{Cdc2}}0^*{-}{\text{Cdc2}}0\left( t \right)} \right| \cdot p_{{{\text{Cdc2}}0}}$$

In addition, we introduce three extra rules:

1. The activation of Clb2_M_ and of Cdc20 in G_2_-M phase of the cell cycle are complex, multi-step processes that incur significant time delays (see, e.g., Fig. 3 of Chen et al.^2^). Therefore, instead of using Eq. (4) for these transitions, $\Delta t$ is assigned a random number drawn from a lognormal(meanlog, sdlog) distribution with meanlog = ${\text{log}}\left( {t_{{{\text{M\_mean}}}} } \right)$ and sdlog = $t_{{{\text{M\_CV}}}}$.
2. When a cell is in G_1_ and Size < *S*_0_, *P*_0_ in Eq. (3) is 0. In this case, no index *j* is selected, and no variable is updated. We set $\Delta t = - {\text{log}}\left( {r_{2} } \right)/p_{{{\text{G1}}}}$, where *r*_2_ is a random number drawn from a uniform distribution between 0 and 1, and *p*_G1_ is the probability of progressing through G_1_ when no variables are changing. During this phase, Size grows according to Eq. (6).
3. When Clb2_G_ changes from 1 to 0, the cell is divided unequally into two cells. The mother cell receives the size equal to
  21 $${\text{Size}}\left( {t + \Delta t} \right) = f \cdot {\text{Size}}\left( t \right) \cdot e^{\mu \cdot \Delta t} ,$$
  where *f* is a random number drawn from a lognormal(meanlog, sdlog) distribution with meanlog = log(*f*_mean_) and sdlog = *f*_CV_. The remaining fraction (1−*f*) belongs to the daughter cell. The rationale behind this rule is explained on p. 375 of Chen et al.^2^

All simulations are carried out using the parameter values in Table 1. With one exception, all propensities are assigned the same value, 1 min^−1^, because we assume that the Boolean switches are fast and we do not want to bias one switch over another during asynchronous updating. The exception, *p*_Cln2_, was set much faster (10 min^−1^) because Cln1/2 are very unstable proteins that quickly switch on and off as SBF switches on and off. The parameters *t*_M_mean_ and *t*_M_CV_ were chosen to match the mean and CV of the budded period *T*_bud_. The parameters *S*_0_mean_, *S*_0_CV_, *f*_mean_ and *f*_CV_ were chosen to match experimental data on *T*_G1_ and its correlation to cell size at birth.

**Table 1:** Parameter values.

Parameter	Value	Parameter	Value
*p*_G1_	1 min^−1^	*μ*	0.007 min^−1^
*p*_Cdh1_	1 min^−1^	*t*_M_mean_	30
*p*_SBF_	1 min^−1^	*t*_M_CV_	0.30
*p*_Cln2_	10 min^−1^	*S*_0_mean_	0.4
*p*_Clb5_	1 min^−1^	*S*_0_CV_	0.05
*p*_Clb2G_	1 min^−1^	*f*_mean_	0.58
*p*_Clb2M_	1 min^−1^	*f*_CV_	0.05
*p*_Cdc20_	1 min^−1^		

## Results

### The logical rules induce a precise order of cell cycle events

Supplementary Table S2 shows all possible updates based on the logical functions in Eqs. (7–13). When these state transitions are connected into a network, we find that all 2^7^ = 128 possible states eventually approach a sequence of 14 recurring states (the cell-cycle ‘highway’), which is the global attractor of the network (Fig. 1b). Starting from any of the 114 states off the highway, the system returns to the highway in three steps, on average (for the full distribution of steps, see Supplementary Fig. S1).

The order of states on the global attractor corresponds to the known sequence of molecular transitions in the budding yeast cell cycle (Table 2). A newborn cell has active Cdh1, while the other variables are inactive (state = 1000000). Once Size grows above a critical size (*S*_0_), SBF is activated with a probability proportional to (Size − *S*_0_)^2^ and the state transitions to (1100000). The rise of SBF promotes the production of Cln2 (1110000), which subsequently turns off Cdh1 (state = 0110000), allowing SBF to promote Clb5 (0111000). Next, Clb5 initiates a two-step activation of Clb2. First, Clb5 activates Clb2_G_ (0111100). Then Clb2_G_ turns off SBF (0011100), and subsequently Cln2 turns off (0001100). Clb2_G_ then activates Clb2_M_ (0001110). It is crucially important that Clb2_G_ turns off Cln2 before it turns on Clb2_M_; otherwise, the sequence of cell cycle events during asynchronous updating can go awry (see Supplementary Text S2). To avoid these problems, we added the condition ‘& ~ Cln2’ to the Boolean Eq. (12) for updating Clb2_M_. We can justify this condition by including the Clb2-kinase inhibitor, Swe1, in our Boolean variable Cln2^46–48^. Swe1 (like Cln1 and Cln2) is synthesized in response to SBF activity, but (unlike Cln1 and Cln2) Swe1 remains active during G_2_ phase until a bud is properly formed. Hence, Clb2_M_ cannot be activated until ‘Cln2’ (i.e., Swe1) is inactivated by Clb2_G_.

**Table 2:** Fourteen states of the cell-cycle highway.

Cdh1	SBF	Cln2	Clb5	Clb2_G_	Clb2_M_	Cdc20	Event (cell cycle phase)
1	0	0	0	0	0	0	Newborn cell (G_1_)
1	1	0	0	0	0	0	SBF turns on (Start)
1	1	1	0	0	0	0	Cln2 turns on (bud emergence)
0	1	1	0	0	0	0	Cdh1 turns off
0	1	1	1	0	0	0	Clb5 turns on (S phase)
0	1	1	1	1	0	0	Clb2_G_ turns on (prophase)
0	0	1	1	1	0	0	SBF turns off
0	0	0	1	1	0	0	Cln2 turns off
0	0	0	1	1	1	0	Clb2_M_ turns on (metaphase)
0	0	0	1	1	1	1	Cdc20 turns on (anaphase)
0	0	0	0	1	1	1	Clb5 turns off
0	0	0	0	1	0	1	Clb2_M_ turns off (telophase)
1	0	0	0	1	0	1	Cdh1 turns on
1	0	0	0	0	0	1	Clb2_G_ turns off (Exit)
1	0	0	0	0	0	0	Cdc20 turns off (G_1_)

The inactivation of Clb2 occurs in two steps during exit from mitosis. First, Clb2_M_ activates Cdc20 (0001111), which in turn inactivates Clb5 (0000111) and Clb2_M_ (0000101). The inactivation of Clb2_M_ allows Cdh1 to be re-activated (1000101) and to shut down Clb2_G_ (1000001). Finally, Cdc20 spontaneously inactivates, and the cell returns to G_1_ (1000000).

Figure 1c shows dynamic activities of Cdh1, Cln2, Clb2 (Clb2_G_ + Clb2_M_), and Cdc20 when the simulation tracks a mother cell, starting in G_1_ phase (1000000) with a cell size of 0.65, through four cycles over the course of ~ 300 min. The cell-cycle transitions follow a precise order of 14 protein states as discussed above and shown in Table 2. The temporal duration of each state, however, is stochastic. Figure 1d shows how the cell grows and divides over this time period. Unlike real mother cells, which increase in volume each generation^34^, in our simulations the ‘Size’ of a mother cell can get smaller from one division to the next, as a consequence of our rule (Eq. 21) for how Size is apportioned between mother and daughter at division. This behavior is acceptable because our ‘Size’ variable is an effective ‘physiological’ size rather than ‘geometric’ size (volume) as measured from micrographs.

In Supplementary Fig. S2, we illustrate how simulated populations of cells grow exponentially with number-doubling time = mass-doubling time (99 min in glucose or 150 min in galactose).

### Population-level statistics in comparison to experiments

To model a population of cells synchronized by size selection, we simulated many newly divided ‘mother’ cells, each starting from G_1_ phase (1000000) with initial Size = 0.65 (the average size of mother cells after division). We then averaged the value of each variable over all simulated cells to represent activity measurements from a population of cells (Fig. 1e,f). The activities of the variables lose synchrony quickly, as observed^34,49^, because of the unequal division of Size between mother and daughter cells.

Supplementary Fig. S3 presents model simulations of a classic experiment by Woldringh et al.^34^ that followed the loss of synchrony in populations of mother and daughter cells during outgrowth from an initial population of size-selected (small) newborn cells. Cell synchrony was assessed by plotting the % budded cells as a function of time. The population of daughter cells (i.e., cells never before divided) loses synchrony rapidly. The cohorts of mother cells with one-, two- or three birth scars show synchronous waves of budding, as observed^34^.

Next, we simulated an asynchronous population of cells by following lineages of mother and daughter cells over many generations and collecting random samples of cells from these simulations. From these samples, we calculated several statistics and compared them to experimental observations. The data include, for both mother and daughter cells,

1. the cell cycle period (*T*_c_ = time from birth to division).
2. the period *T*_G1_ from mitotic exit (state 1000001) to SBF activation (state 1100000), which corresponds experimentally to the interval between cell separation and bud emergence^17^.
3. the period *T*_bud_ from bud emergence (state 1100000) until the end of the cycle (1000001).
4. cell size at birth.

**Figure 2:**
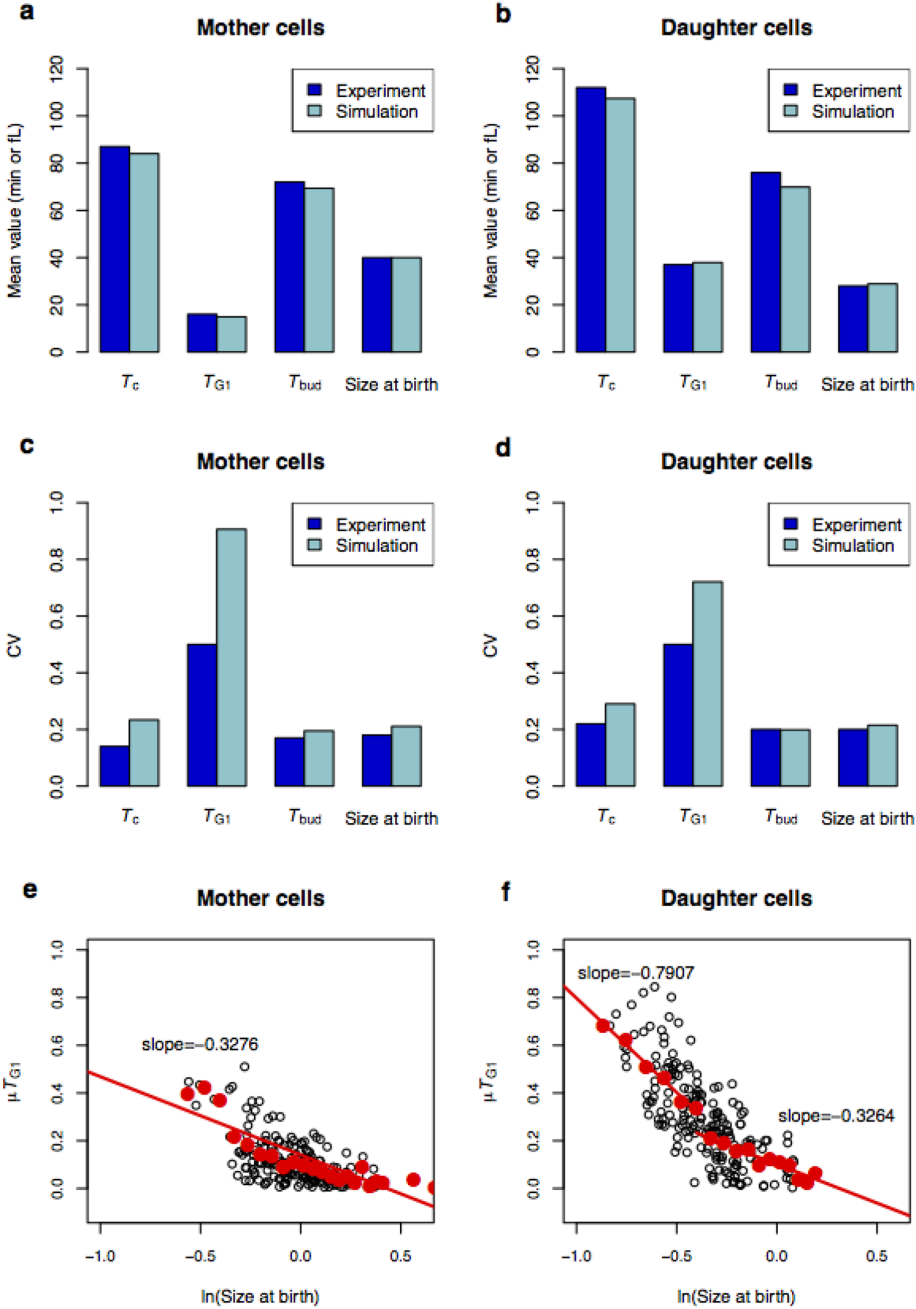
Statistical properties of cell-cycle attributes calculated for simulated populations of unsynchronized mother and daughter cells. Mean values (**a** and **b**) and coefficients of variation (**c** and **d**) of cell cycle durations and size at birth: *T*_c_ = cell cycle period (from birth to division), *T*_G1_ = duration of unbudded phase (from birth to SBF activation), *T*_bud_ = duration of budded phase (from SBF activation to cell division). Model predictions (teal) are compared to experimental observations^17^ (blue). To compare the experimental mean-value of Size-at-birth (volume, fL) to the dimensionless Size-at-birth in our model, we assume a conversion factor of 61.4 fL, which aligns the mean values for mother cells but not for daughter cells. The CV’s are not affected by this assumption. (**e** and **f**) Joint distributions between Size-at-birth and G_1_ duration (*T*_G1_). Size-at-birth is measured relative to the average size of mother cells at birth. Two hundred mother and daughter cells (black open circles) were sampled randomly from the whole population. Large red dots represent the average values of μT_G1_ of the black open circles binned in 2 fL intervals, as was done for the experimental data in DiTalia et al.^17^ Except for the smallest mother cells, the binned data can be fitted by a single straight line of slope − 0.33. For daughter cells, the binned data is fitted by two straight lines of slope = − 0.33 for large daughter cells and slope = − 0.79 for small daughter cells. The experimental slopes^17^ are − 0.1, − 0.3, and − 0.7 for mother, large daughter, and small daughter cells, respectively. In Supplementary Fig. S4 we show the results of all ~ 1500 simulated data points.

As Fig. 2a–d shows, the statistics computed from the model are in good agreement with experiments^17^, except CV of the G_1_ period (*T*_G1_). Clearly, the model overestimates the variability in progression through G_1_, despite the fact that size control in the Boolean model is implemented analogous to size control in our stochastic ODE model^50^, which provides a good fit to the mean and CV of *T*_G1_ in both mother and daughter cells (see Fig. 10 of Laomettachit et al.^50^). (We haven’t yet tracked down the cause of this discrepancy.) Finally, in Fig. 2e,f we plot the joint distributions between Size-at-birth and *T*_G1_ for mother cells and daughter cells and compare them to experiments in Di Talia et al.^17^ In Fig. 2e,f we plot the correlations for ~ 200 cells, comparable to the number of cells in the experimental data set. (For the complete set of ~ 1500 simulations, see Supplementary Fig. S4.) Perfect ‘size control’ of cell entry into G_1_ would correspond to a correlation coefficient = − 1 for *µT*_G1_ in dependence on ln(Size-at-birth). Using the same ‘binning’ method proposed by Di Talia et al., we compute correlation coefficients of − 0.33 for mother cells and − 0.79 (− 0.33) for small (large) daughter cells, which are in good agreement with experimental slopes of − 0.1, − 0.7 and − 0.3, respectively^17^. For a thorough statistical analysis of the experimental data, see Supplementary Text S2 in Di Talia et al.^17^.

### Cell cycle progression at slower growth rates

When budding yeast cells are grown on a poor carbon source (galactose or ethanol), they are generally smaller than cells grown on glucose, and they exhibit a longer interdivision time, primarily because of a lengthened unbudded interval^51^. To model these effects, we simulated yeast cells growing with mass-doubling time of 150 min (Supplementary Fig. S5). Compared to Fig. 1f, these simulated cells are indeed smaller, and they lose synchrony more quickly than cells grown on glucose (*mdt* = 99 min). From many such simulations, we computed statistics for mother and daughter cells (Supplementary Fig. S6). Mother cells had an average cycle time of 117 min, and daughter cells 183 min. For cells growing in glycerol (*mdt* = 152 min), Lord & Wheals^51^ observed average cycle times of 118 min and 188 min for mother and daughter cells, respectively. For cells growing in raffinose (*mdt* = 155 min), Ball et al.^52^ observed a lesser spread between cycle times of mother (132 min) and daughter (160 min) cells.

### Robust recovery from perturbations

We analyzed all non-trivial, single-state perturbations of the Boolean variables during normal progression through the 14 states of the cell-cycle highway. Each of the 14 states has seven components, one of which was updated from the previous state and another one is to be updated in the next state; so five of the components are subject to perturbation away from the highway, by flipping from 0 to 1 or vice versa. Hence, there are 14 × 5 = 70 single-state perturbations to consider. For example, suppose we perturb State 6 = 0111100 (Clb2_G_ has just turned on) by flipping Cdc20 (component 7) from 0 to 1. We then follow all possible sequences of states until the system reaches the stable sink G_1_ = 1000000, and we check to see if the perturbed sequence of events is ‘normal’ or ‘aberrant’. In this case, in 77% of the sequences, the simulated cell divides before activating Clb2_M_, i.e., before aligning replicated chromosomes on the mitotic spindle, which would produce aneuploid (inviable) progeny.

For each of the 70 perturbation simulations, cell-cycle progression is considered normal if the following events occur in the correct order:

1. DNA replication (either Clb5 or Clb2_G_ turns on),
2. Prophase entry (Clb2_G_ turns on),
3. Proper spindle fiber attachment (Clb2_M_ turns on),
4. Sister chromatid segregation (Cdc20 turns on), and.
5. Mitotic Exit (Clb2_G_ turns off).

In addition, a bud must emerge (either Cln2 or Clb5 turns on) before mitotic entry. Aberrant progressions are classified according to the problem encountered.

Supplementary Table S3 shows the results of 5000 simulations for each of the 70 single-state perturbations. Out of these 350,000 simulations, 318,172 (91%) robustly resumed the normal cell-cycle sequence of events. The 9% of aberrant sequences were from the 12 perturbations listed in Table 3, which can be categorized into three patterns.

**Table 3:** ‘Single-state’ perturbations that lead to aberrant cell-cycle sequences.

State of perturbation	Cell-cycle phase of perturbation	Variable perturbed	Resuming normal progression (%)	Resulting in an abnormal sequence (%)
1,000,000	G_1_	Clb2_G_ turns on	50.40	49.60^b^
		Clb2_M_ turns on^a^	16.50	83.50^b^
1,100,000	Start	Clb2_G_ turns on	50.16	49.84^b^
		Clb2_M_ turns on^a^	16.22	83.78^b^
0111000	S	Cdc20 turns on	73.52	26.48^c^
0111100	Prophase	Cdh1 turns on	49.48	50.52^d^
		Cdc20 turns on	23.04	76.96^c^
0011100	Prophase	Cdh1 turns on	49.98	50.02^d^
		Cdc20 turns on	46.48	53.52^c^
0001100	Prophase	Cdh1 turns on	49.72	50.28^d^
		Cdc20 turns on	50.82	49.18^c^
0001110	Metaphase	Cdh1 turns on	87.12	12.88^d^

For each perturbation, the percentages are calculated from 5000 repeats.

^a^When Clb2_M_ is flipped on, Clb2_G_ is also set to be on.

^b^Exit without bud emergence.

^c^Exit without activating Clb2_M_.

^d^Exit without activating Cdc20.

**Figure 3:**
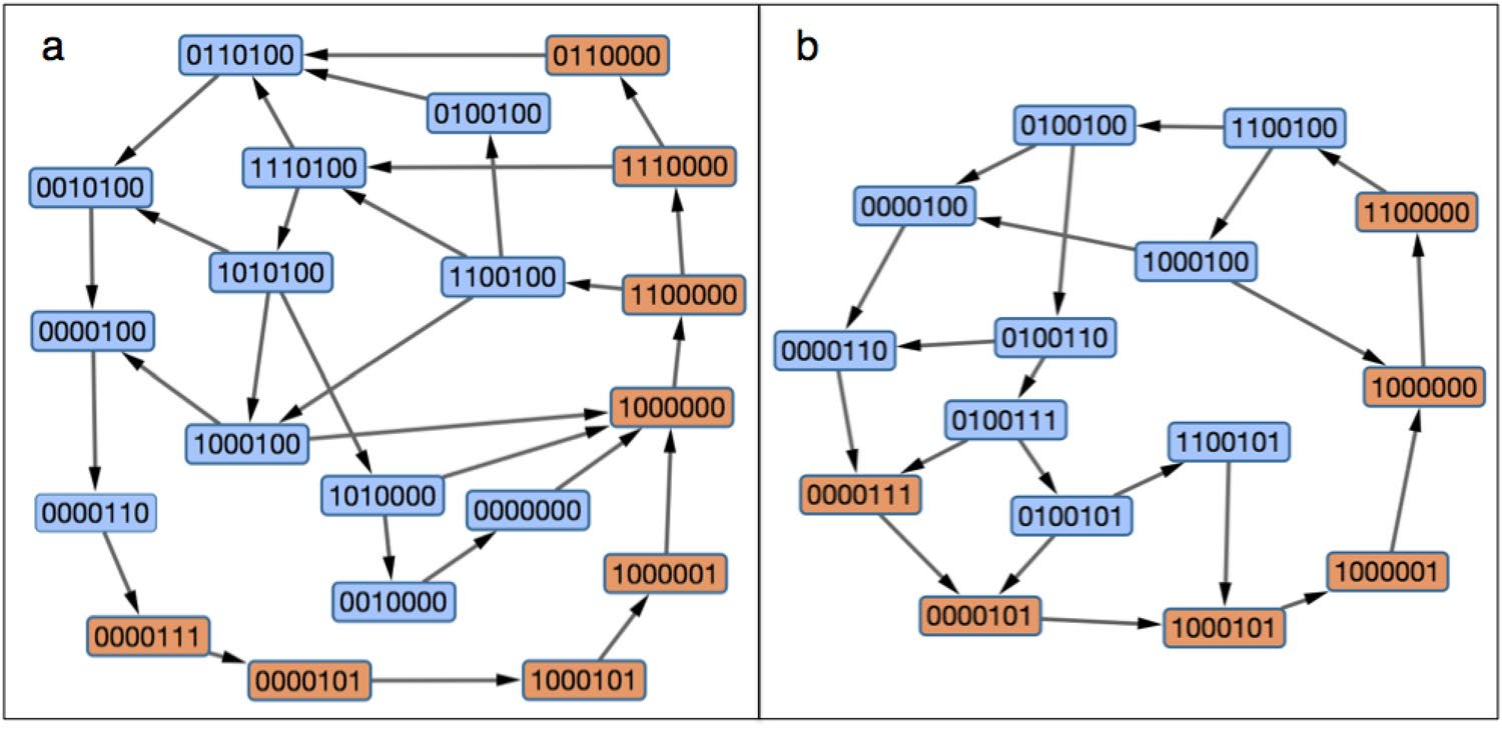
Simulations of mutant strains with a single B-type cyclin (Clb2), as described by Pirincci Ercan et al.^55^ (**a**) Clns-Clb2^S-M^. (**b**) *MET3pr*-Cln2-Clb2^G1-S-M^. In each case, we simulated 500 cells starting in G_1_ (1000000) until they returned to G_1_ and then constructed networks from the union of the 500 trajectories. Orange (blue) boxes denote states on (not on) the cell cycle highway.

First, Clb2_G_ or Clb2_M_ is pre-maturely activated during two early states (1000000 and 1100000). In these cases, either Clb2 is immediately inactivated by Cdh1 or Cdh1 is inactivated by Clb2. In the former case (33% of 20,000 simulations), the cells resume normal cell-cycle progression. In the latter case (67%), however, the cells proceed to activate Cdc20 and exit mitosis without budding (Cln2 and Clb5 never turn on or they turn on after cells enter prophase).

Second, Cdc20 is activated pre-maturely during four states corresponding to S phase and prophase (0111000, 0111100, 0011100, and 0001100). These simulations resemble a defective anaphase checkpoint (i.e., Cdc20 is activated before metaphase). From 20,000 simulations of those four states, 52% proceed to mitotic exit without activating Clb2_M_. These cells exit mitosis without proper metaphase alignment of chromosomes and produce progeny with DNA copy-number abnormalities.

Third, Cdh1 is activated prematurely during four states corresponding to prophase to metaphase (0111100, 0011100, 0001100, and 0001110). In 41% of 20,000 simulations, the cells divide without activating Cdc20 (no segregation of replicated chromosomes) and produce progeny with DNA copy-number abnormalities.

### Mutant simulations

A hallmark of detailed ODE simulations of the budding yeast cell cycle^1,50,53^ is their ability to simulate accurately the phenotypes of hundreds of mutant strains. Of course, simple Boolean models, like Li et al.’s and ours, cannot possibly compete because they do not account for all the gene products that are perturbed in the mutant strains, nor for the redundancies among the regulatory proteins. For example, knocking out any component (setting *B*_*i*_ = 0) is lethal in these models but not in reality.

We can easily simulate Δ*cln3* mutant cells, which are about 70% larger than wild-type cells^54^, by increasing *S*_0_mean_, the minimum size requirement for executing Start. The results are displayed in Supplementary Fig. S7.

A more challenging test is to simulate two interesting mutant strains recently studied by Pirincci Ercan et al.^55^.

- Clns-Clb2^S-M^ strain (has Clns1/2/3, lacks Clbs5/6, *CLB2* expressed from *CLB2* promoter and from *CLB5* promoter): viable; bud emergence normal, DNA synthesis delayed.
- *MET3pr*Cln2-Clb2^G1-S-M^ strain (lacks Clns1/3 and Sic1, *CLN2* expression repressible by methionine, lacks Clbs5/6, *CLB2* expressed from *CLN2*, *CLB5* and *CLB2* promoters): inviable in methionine, no bud emergence, DNA replication and mitosis in mother cell, no cell division.

To simulate these mutants with our BKMC model, we made the following changes demanded by the mutant genotypes:

- Clns-Clb2^S-M^ strain: Clb5* = 0, Clb2_G_* = Clb2_M_ | ( ( Clb5 | Clb2_G_ ) & ~ Cdh1 ) | SBF.
- *MET3pr*Cln2-Clb2^G1-S-M^ strain in methionine: additional changes Cln2* = 0, *S*_0_mean_ = 0.8 (100% increase) because Δ*cln3*.

In each case we simulated 500 cells starting at state (1000000) until they returned there by a variety of trajectories (Fig. 3). In the first case (Fig. 3a), after SBF is activated, either Cln2 or Clb2_G_ is turned on. In the majority of trajectories (431/500), Cdh1 turns off (1 → 0 in the first digit) with Clb2_G_ on (1 in the fifth digit); consequently, Clb2_M_ turns on, then Cdc20 turns on, and the cell exits mitosis and returns to G_1_. So, the majority of cells go through a viable sequence of cell-cycle states. DNA synthesis may be delayed if Clb2 is not as effective as Clb5 in initiating S phase. In a minority of trajectories (69/500), Cdh1 stays on (1 in the first digit) and turns Clb2_G_ off, so the cell returns to G_1_ (1000000) and tries again. In either case, the cells are viable, with some delay in S phase. In the second case (Fig. 3b), after Clb2_G_ is turned on by SBF (remember, Cln2 is always off in methionine), there are two options: (1) Cdh1 kills Clb2_G_ (135/500) and the cell returns to G_1_ and tries again, or (2) Clb2_G_ kills Cdh1 (365/500) and activates Clb2_M_, followed by Cdc20 activation and exit from mitosis, a more-or-less normal sequence of cell cycle events. However, because the cell never made a bud, it cannot divide, which is the observed phenotype.

## Discussion

We have investigated a continuous-time discrete-state Boolean model of the cell-cycle control system of budding yeast, using the Boolean Kinetic Monte-Carlo (BKMC) approach^15^. Inspired by Gillespie’s stochastic simulation algorithm^33^, the Boolean state (a binary seven-digit number) and the simulation time (a real number) are updated stochastically. The logical rules governing updates of the Boolean variables reveal that all 128 possible initial states are attracted to a 14-state ‘cell-cycle highway’ that corresponds to normal progression through major cell cycle phases (late G_1_ → S → G_2_ → M → CD → early G_1_). Adding a simple rule for a growth-controlled Start transition (early G_1_ → late G_1_, by activation of the transcription factors SBF and MBF), we are able to simulate the proliferation of mother and daughter cells in an exponentially expanding population of budding yeast cells. From these simulated populations we computed several statistical properties of cell cycle progression, and they are in good agreement with experimental observations (Fig. 2).

We can also use the model to explore the ‘robustness’ of cell-cycle progression with respect to perturbations away from the cell-cycle highway. Because the highway is globally attracting (Fig. 1b), any perturbation will eventually return, but the sequence of cell cycle events during the return may be abnormal and potentially lethal. Considering all nearby perturbations from the cell-cycle highway (i.e., the 70 initial states that differ from the highway by flipping one switch from *B*_*i*_ to ~ *B*_*i*_), we find that 91% of the perturbations return to the normal cell-cycle sequence of events without difficulties, but 9% induce abnormal sequences, such as aberrant partitioning of chromosomes at mitosis, or division without bud formation (Table 3). The abnormal sequences are the results of drastic perturbations: (1) activation of mitotic cyclins (Clb1, Clb2) in G_1_ phase, before bud emergence; (2) activation of exit proteins (Cdc20, Cdc14) in S or G_2_ phase, before the mitotic spindle can be assembled; and (3) activation of Cdh1 and Sic1 (cell division) in prophase or metaphase, before Cdc20 can induce anaphase separation of replicated chromosomes. All these perturbations can be expected to have lethal consequences.

Despite the fact that the model, with only seven Boolean variables + cell size (a continuous variable), is greatly oversimplified, it conforms in quantitative detail to many experimental observations of the growth and division of wild-type cells. The next logical step would be to extend the model by separating the ‘lumped’ variables (SBF/MBF, Cdh1/Sic1, Cdc10/Cdc14) and to include other crucial regulatory proteins (e.g., Whi5, Mcm1, Swi5, Cdc5; see Fig. 8 in Laomettachit et al.^50^), whose roles are necessary to understand the phenotypic properties of mutant strains of budding yeast. Success in this endeavor will require a careful consideration of the logical rules describing protein interactions and a disciplined effort to optimize parameters over a range of experimental data types. It will be interesting to see if a more complete BKMC model of the budding yeast cell cycle can be as successful as comprehensive nonlinear ODE models in giving a quantitative account of mutant phenotypes. If so, then BKMC modeling may prove competitive with ODE modeling, which is more computationally demanding in terms of simulation time and parameter estimation.

## Supplementary Information

Supplementary Information.

## Data Availability

All computer codes are available on Github: https://github.com/pribnowbox/a-continuous-time-stochastic-Boolean-model-of-the-budding-yeast-cell-cycle.
