# Supplementary material for: A continuous-time stochastic Boolean model provides a quantitative description of the budding yeast cell cycle
Source: Sci Rep. 2022 Nov 24;12:20302. doi: 10.1038/s41598-022-24302-6 (PMC9700812; doi:10.1038/s41598-022-24302-6)
Supplement: Supplementary file 1 — Supplementary Information. [file 41598_2022_24302_MOESM1_ESM.pdf]

Supplementary Information

A continuous-time stochastic Boolean model provides a quantitative description of the budding yeast cell cycle

Teeraphan Laomettachit, Pavel Kraikivski and John J. Tyson

Supplementary Text S1. Robustness of Li et al. model under asynchronous updating.

In Li's model, the cell cycle follows a sequence of 13 states and returns to a fixed point corresponding to a stationary G_1 cell, as shown in the following Table A (reproduced from Li et al.[1]).

Table A. The 13 cell-cycle states in Li's model[1].

State	Cln3	MBF	SBF	Cln1,2	Cdh1	Swi5	Cdc14,20	Clb5,6	Sic1	Clb1,2	Mcm1
1	1	0	0	0	1	0	0	0	1	0	0
2	0	1	1	0	1	0	0	0	1	0	0
3	0	1	1	1	1	0	0	0	1	0	0
4	0	1	1	1	0	0	0	0	0	0	0
5	0	1	1	1	0	0	0	1	0	0	0
6	0	1	1	1	0	0	0	1	0	1	1
7	0	0	0	1	0	0	1	1	0	1	1
8	0	0	0	0	0	1	1	0	0	1	1
9	0	0	0	0	0	1	1	0	1	1	1
10	0	0	0	0	0	1	1	0	1	0	1
11	0	0	0	0	1	1	1	0	1	0	0
12	0	0	0	0	1	1	0	0	1	0	0
13	0	0	0	0	1	0	0	0	1	0	0

The model used the updating functions

$$S_{t+1}(x) = \begin{cases} 1, x > 0 \\ 0, x < 0 \\ S_t, x = 0 \end{cases}$$

$$S'_{t+1}(x) = \begin{cases} 1, x > 0 \\ 0, x \leq 0 \end{cases}$$

where the original Boolean functions for the 11 variables are:

$\text{Cln3}_{t+1} = S'(\text{Size}_t)$

$\text{MBF}_{t+1} = S(\text{Cln3}_t - \text{Clb1,2}_t)$

$\text{SBF}_{t+1} = S(\text{Cln3}_t - \text{Clb1,2}_t)$

$\text{Cln1,2}_{t+1} = S'(\text{SBF}_t)$

$\text{Cdh1}_{t+1} = S(\text{Cdc14,20}_t - \text{Cln1,2}_t - \text{Clb5,6}_t - \text{Clb1,2}_t)$

$\text{Swi5}_{t+1} = S'(\text{Mcm1}_t + \text{Cdc14,20}_t - \text{Clb1,2}_t)$

$\text{Cdc14,20}_{t+1} = S'(\text{Mcm1}_t + \text{Clb1,2}_t)$

$\text{Clb5,6}_{t+1} = S(\ \text{MBF}_t - \text{Sic1}_t - \text{Cdc14,20}_t\ )$

$\text{Sic1}_{t+1} = S(\ \text{Cdc14,20}_t + \text{Swi5}_t - \text{Cln1,2}_t - \text{Clb5,6}_t - \text{Clb1,2}_t\ )$

$\text{Clb1,2}_{t+1} = S(\ \text{Mcm1}_t + \text{Clb5,6}_t - \text{Sic1}_t - \text{Cdh1}_t - \text{Cdc1420}_t\ )$

$\text{Mcm1}_{t+1} = S'(\ \text{Clb5,6}_t + \text{Clb1,2}_t\ )$

The variables were updated synchronously in Li's model, i.e., all variables that are scheduled to change state do so simultaneously. Indeed, two or three variables simultaneously change their activities in many transitions (see Table A above).

We are interested in exploring Li's model with asynchronous updating. Our aim is to see
1) if sequences of asynchronous updates follow or remain close to the 13 cell-cycle states and return to the stationary G_1 fixed point or deviate far from the 13 states and reach different fixed points, and
2) if, during asynchronous updating, the major events of the cell cycle execute in the proper order: Cln1,2 turns on (bud emergence); Clb5,6 turns on (DNA replication); Clb1,2 turns on (mitotic entry); Cdc14,20 turns on (sister chromatid separation); Clb1,2 turns off (mitotic exit).

We modified Li's model to update asynchronously (i.e., only one variable changes its value from t to $t+1$, where the variable to change is chosen randomly). Based on 5000 simulation repeats starting from 'State 1' (10001000100), 56.60% finally reached stationary G_1 (00001000100). The other 44.40% ran off to one of the other five fixed points of the model, as shown in the following table.

Table B. Fixed points found in Li's model with asynchronous updating.

Fixed point	Number of trajectories reaching the fixed point	Percentage
00001000100 (stationary G_1)	2830	56.60%
00110000000	905	18.10%
01001000100	820	16.40%
00000000100	423	8.46%
00000000000	15	0.30%
00001000000	7	0.14%

In addition, we noted if the major cell-cycle events are executed in the correct sequence when the model is updated asynchronously. Based on 5000 simulation repeats, four patterns were observed.

1. 1195 repeats exhibited normal cell cycle progression, as defined above.
2. 445 repeats exhibited pre-mature activation of Cdc14,20 resulting in 'mitotic catastrophe': Cln1,2 turns on (bud emergence), Clb5,6 turns on (DNA replication), Cdc14,20 turns on, i.e., degradation of the 'cohesin' proteins that hold sister chromatids together before Clb1,2 turns on (mitotic spindle formation) and Clb1,2 turns off (mitotic exit). This 'catastrophe' happens because the original rule allows Cdc14,20 to be activated by Mcm1 before activation of Clb1,2, a mistake that does not happen with synchronous updating because Mcm1 and Clb1,2 turn on

simultaneously. However, with random asynchronous updating, the following order is possible: Mcm1 turns on; Cdc14,20 turns on; Clb1,2 turns on.

3. 894 repeats executed bud emergence (Cln2 turns on) and then arrested in state '00110000000'. In this sequence of events, Cln3 stochastically turns off after it activates SBF, which then activates Cln2 (bud emergence); however, MBF remains off, and the cell cycle is arrested without turning on Clb5,6 or Clb1,2.
4. 2466 repeats did not execute any major events because: either Cln3 turns off immediately and the cell reverts to stationary G_1 '00001000100', or Cln3 turns off after activating MBF but before SBF is activated and the cell arrests in state '01001000100'.

We can fix these problems by two reasonable modifications of the Boolean rules of Li's model:

First, after Cln3 turns on in State 1 (see Table A), Cln3 stays on during the entire cell cycle progression until Clb1,2 turns off at cell division (State 10 in Table A). In addition, the rules for MBF and SBF are changed from $S(\text{Cln3}_t - \text{Clb1,2}_t)$ to $S'(\text{Cln3}_t - \text{Clb1,2}_t)$, to allow Clb1,2 to turn off SBF and MBF even when Cln3 is on. The modification eliminates Problem Patterns 3 and 4 by allowing SBF and MBF to be regulated properly during cell-cycle progression. This modification also removes two fixed points ('00110000000' and '01001000100').

Second, we resolve Problem Pattern 2 (premature Cdc14,20 activation before Clb1,2) by changing the original rule $\text{Cdc14,20}_{t+1} = S'(\text{Mcm1}_t + \text{Clb1,2}_t)$ to $\text{Cdc14,20}_{t+1} = S'(\text{Clb1,2}_t)$.

With these changes, all 5000 repeats exhibit the correct sequence of cell-cycle events. Although the problems that arise when the original model is updated asynchronously are easily fixed by modifying some of the Boolean rules, the results indicate that cell-cycle 'robustness' depends sensitively on the rules and the updating scheme. In particular, asynchronous updating can dramatically disrupt the robustness exhibited under synchronous updating.

Supplementary Text S2. Problems arise when Clb2$_M$ turns on before Cln2 turns off.

If we allow Clb2$_M$ to turn ON before Cln2 turns OFF, then the 14-state ‘cell-cycle highway’ (the orange states, below) expands to 22 states, encompassing alternative routes (the blue sequences, below) from early G$_2$ (state 0111100) to telophase (0000101). These alternative routes involve SBF turning OFF and Cln2 turning OFF later and later in the cycle: sequences that are never observed in budding yeast division cycles. To disallow these alternative sequences, we require that Cln2 turn OFF before Clb2M can turn ON.

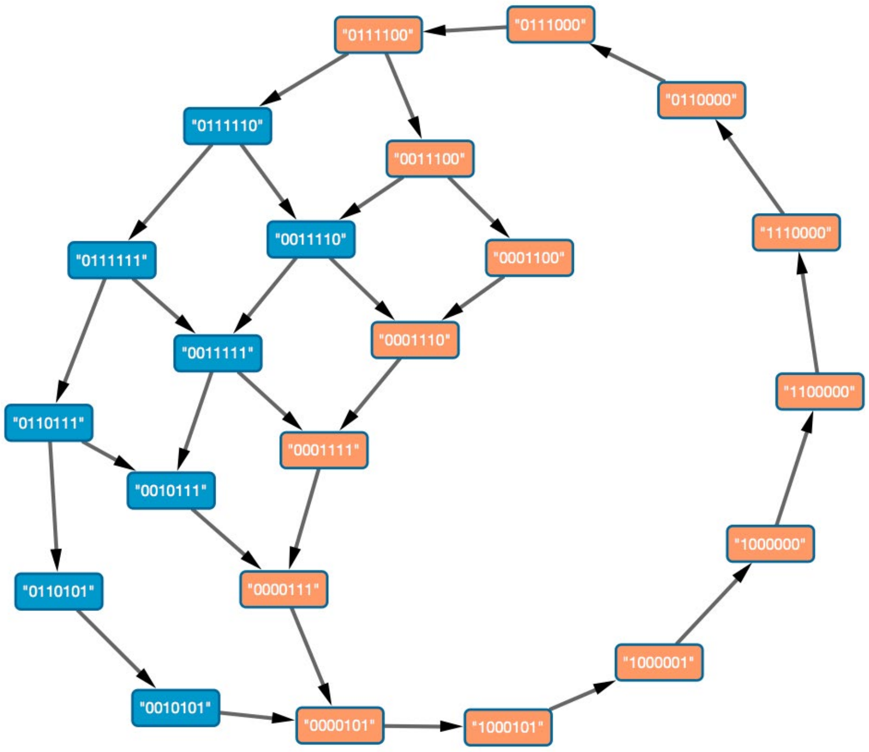

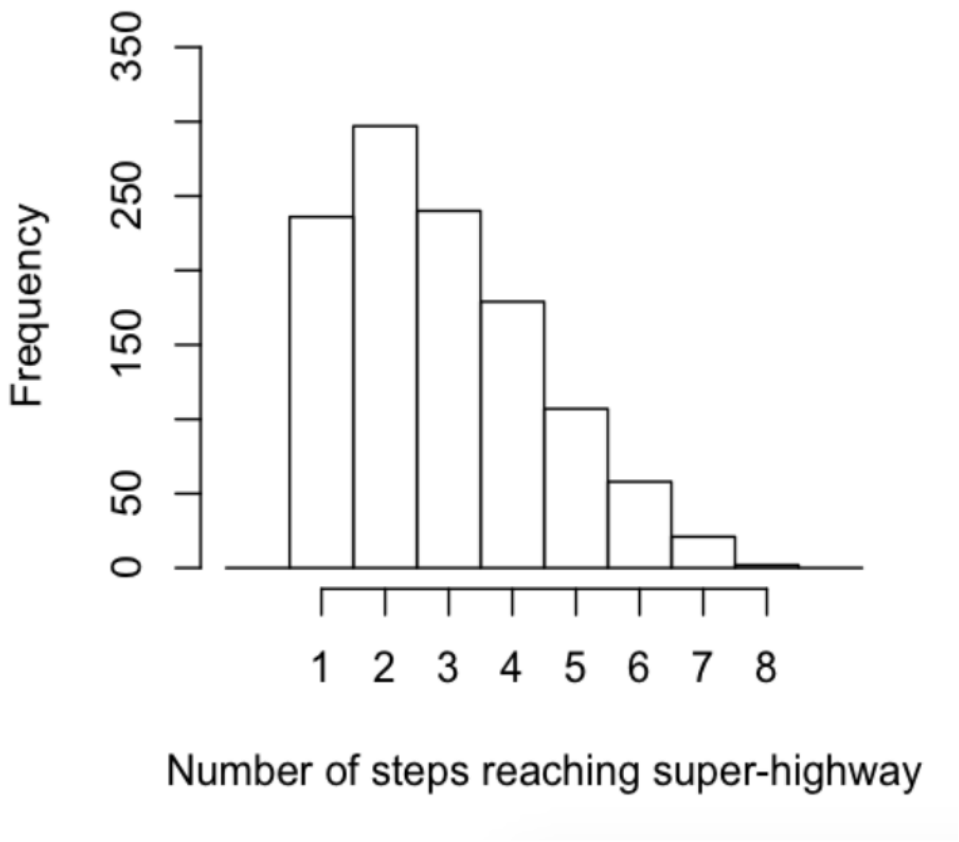

Supplementary Figure S1. Frequency distribution for re-entry into the cell-cycle highway. Starting from each of the 114 states off the cell-cycle highway (the large circle of states in Fig. 1b), we follow ten sample trajectories until the protein state reaches one of the 'highway' states (the 14 states off the circle in Fig. 1b). From these 1140 simulations, we plot a histogram of the number of steps taken to reach the highway (average # steps = 2.9).

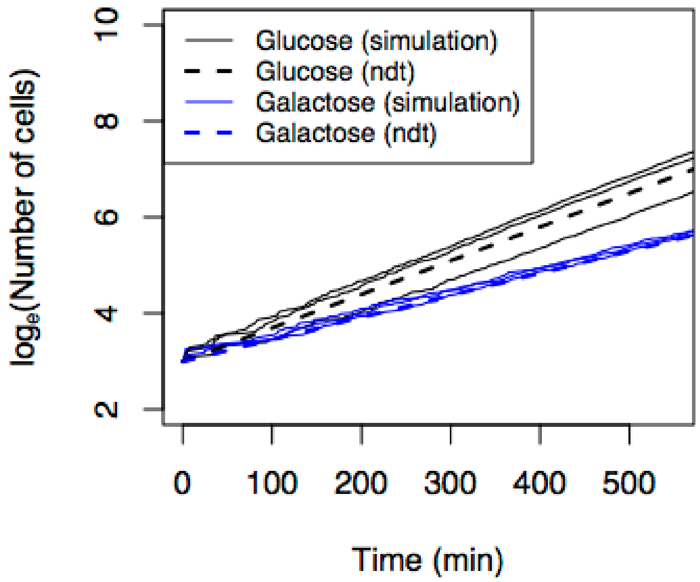

Supplementary Figure S2. Log_e(number of cells) grown on glucose (black, *mdt* = 99 min) and galactose (blue, *mdt* = 150 min). Solid lines, three independent simulations (starting from 20 cells) for each carbon source; dashed lines, expected increase in cell number = $20 \times 2^{t/mdt}$. In each simulation, 10 mother and 10 daughter cells were followed for 2000 min to create an asynchronous population of 10 mother and 10 daughter cells. Then, starting from these 20 cells, the full lineages of mother and daughter cells were simulated for 600 min and the total number of cells was plotted as a function of time.

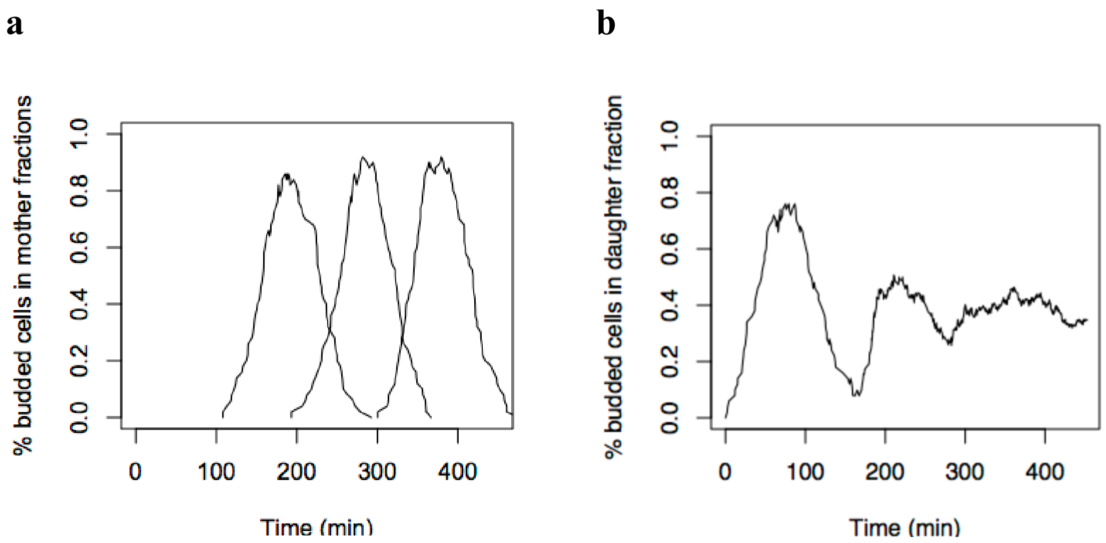

Supplementary Figure S3. Loss of synchrony during cell proliferation. (**a**) Fraction of budded mother cells with one-, two- and three birth scars. (**b**) Fraction of all daughter cells (i.e., unscarred cells) extant at time *t* that are budded. An initial population of 50 small daughter cells (Size = 0.45) was followed until they all bud and divide, which produces 50 mother cells with one scar. The 50 mother cells with one scar were followed until they all bud and divide (first peak in panel a), then the 50 mother cells with two scars were followed (second peak), and the 50 mother cells with three scars were followed (third peak). In panel b, all daughter cells (the initial population plus the daughters produced by all mother cells) were monitored over time, and the fraction carrying buds was plotted as a function of *t*. These plots should be compared to Figure 5 of Woldringh et al.[2] In this simulation, *mdt* = 120 min, as is the case in the experimental population.

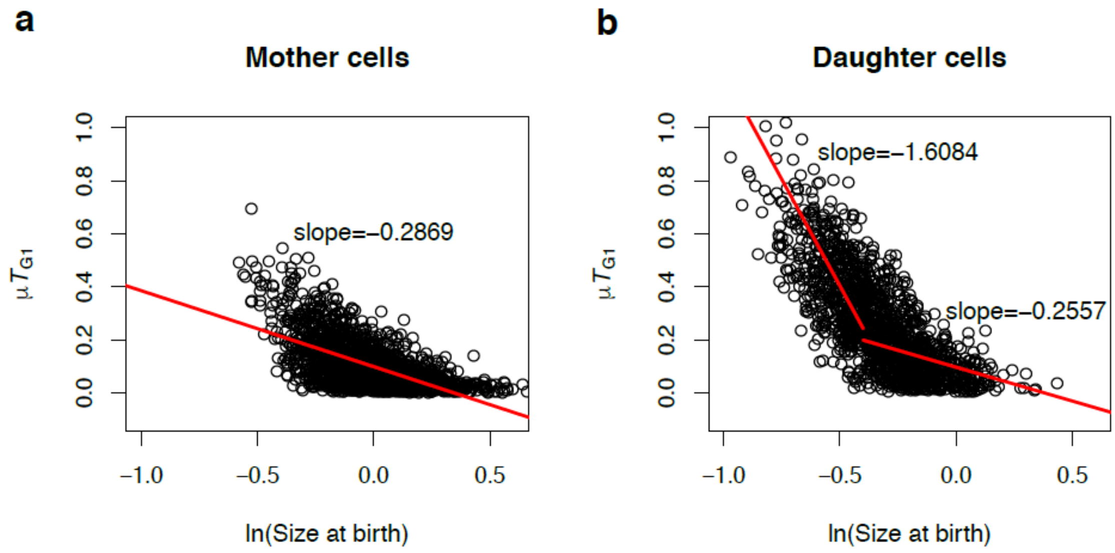

Supplementary Figure S4. Joint distributions between Size-at-birth and G_1 duration (T_{G1}) for 1669 mother cells (**a**) and 1391 daughter cells (**b**). Size-at-birth is measured relative to the average size of mother cells at birth. The mother cell distribution is fitted to a straight line of slope −0.287. The daughter cell is fitted to two straight lines of slope = −0.256 and −1.61.

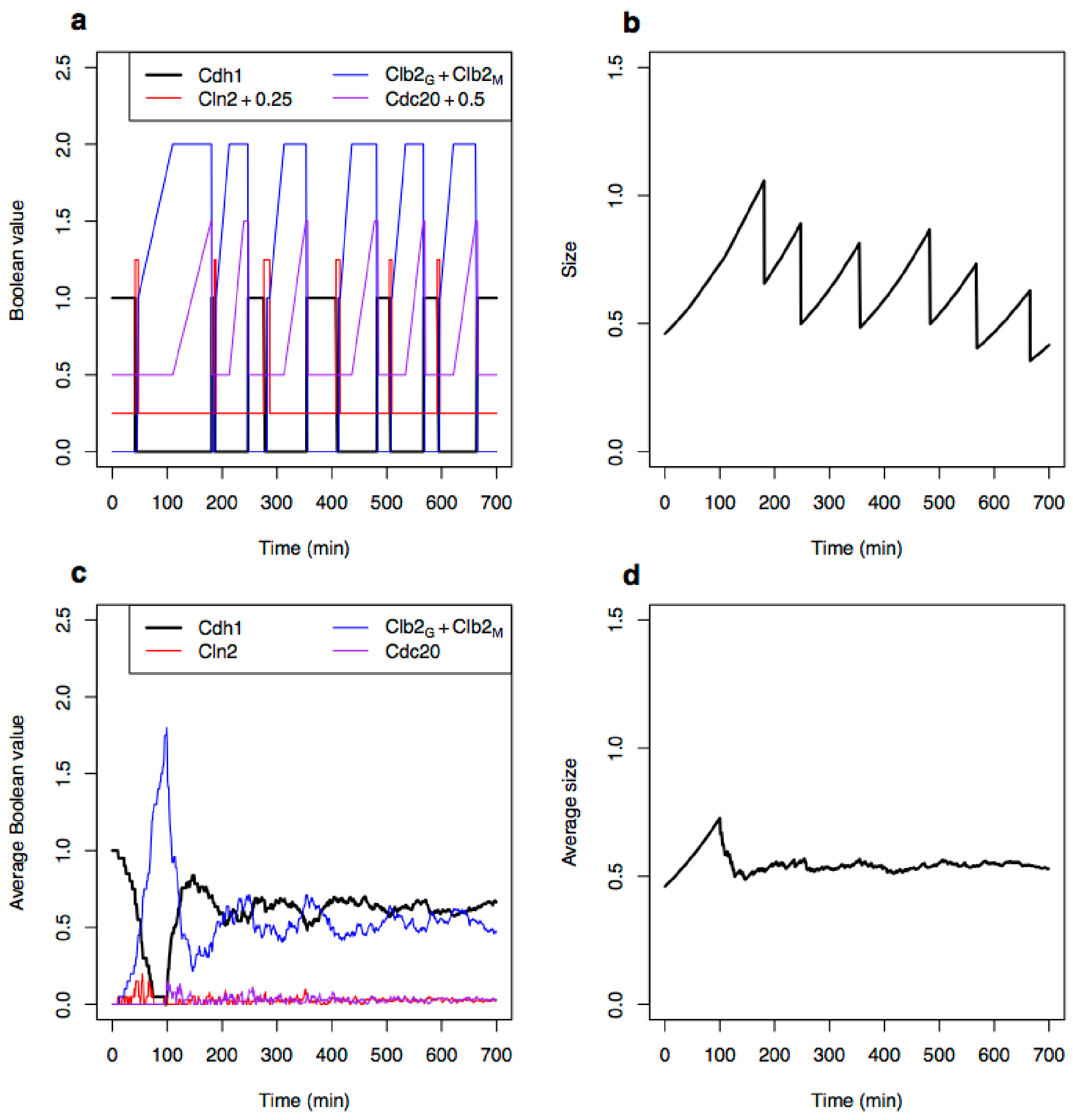

Supplementary Figure S5. Simulated budding yeast cells grown in galactose. To account for the slower growth rate (*mdt* = 150 min) on the medium, μ is set to 0.0046 min^{-1}. Time evolution of Boolean variables (**a**) and cell size (**b**) from a single-cell simulation. (**c** and **d**) Values of the same variables, averaged over a population of initially size-selected cells. A population is started from 20 cells of Size = 0.46 (the average size at birth of mother cells in galactose), which are followed along with their progeny (both mother and daughter cells) for 700 min. Averages are calculated over all cells extant at time *t*.

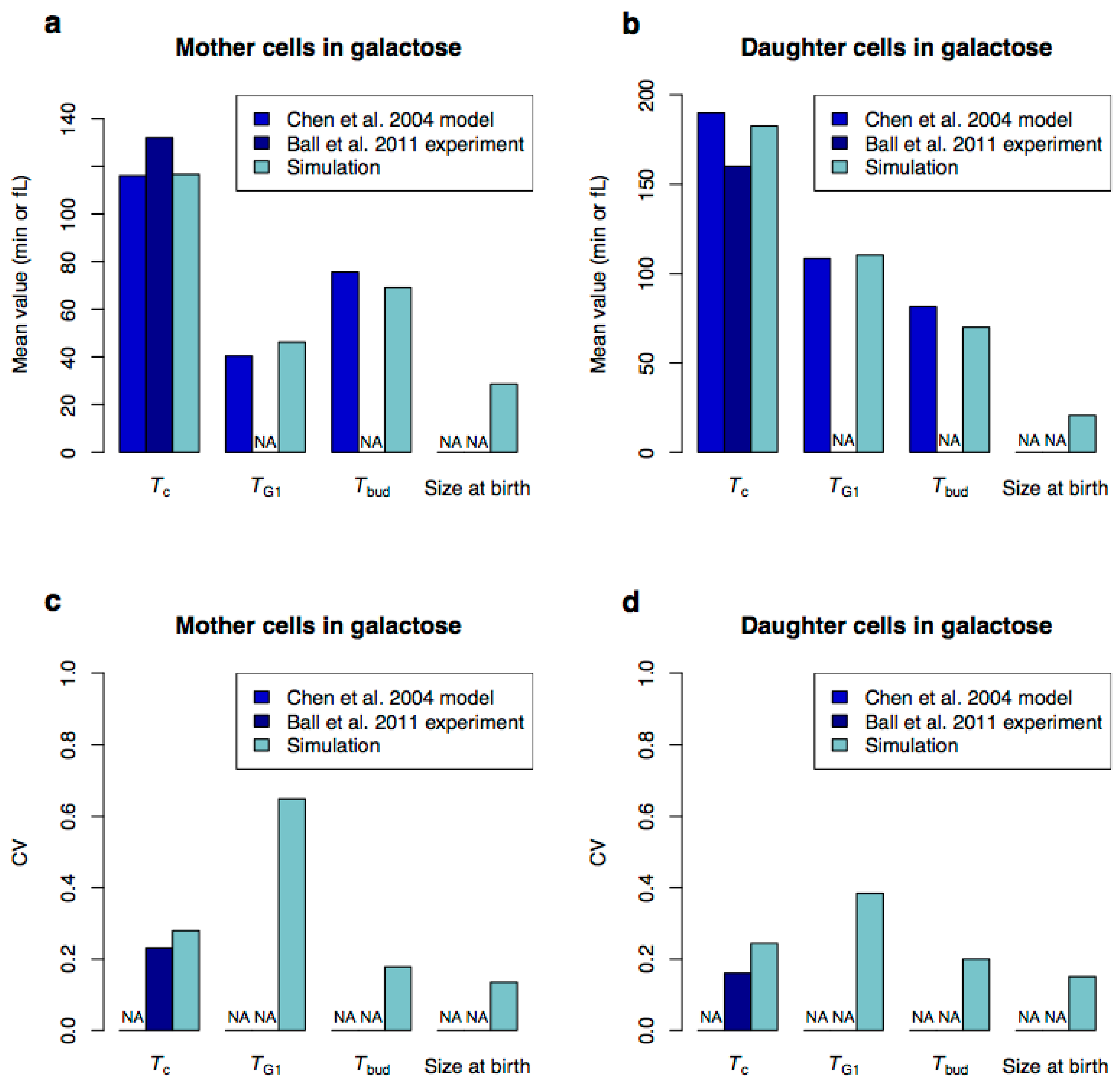

Supplementary Figure S6. Statistical properties of cell-cycle attributes calculated for simulated populations of unsynchronized mother and daughter cells grown in galactose (mdt = 150 min, μ = 0.0046 min^{-1}). Our simulations results are compared to the simulations of Chen et al.[3] and to experimental results reported in Table 1 (Experiment 1, wild-type cells) in Ball et al.[4] Dimensionless Size-at-birth in our model is converted to fL by a constant multiple, 61.4 fL, the same value used for the cells grown in glucose. NA means data is not available.

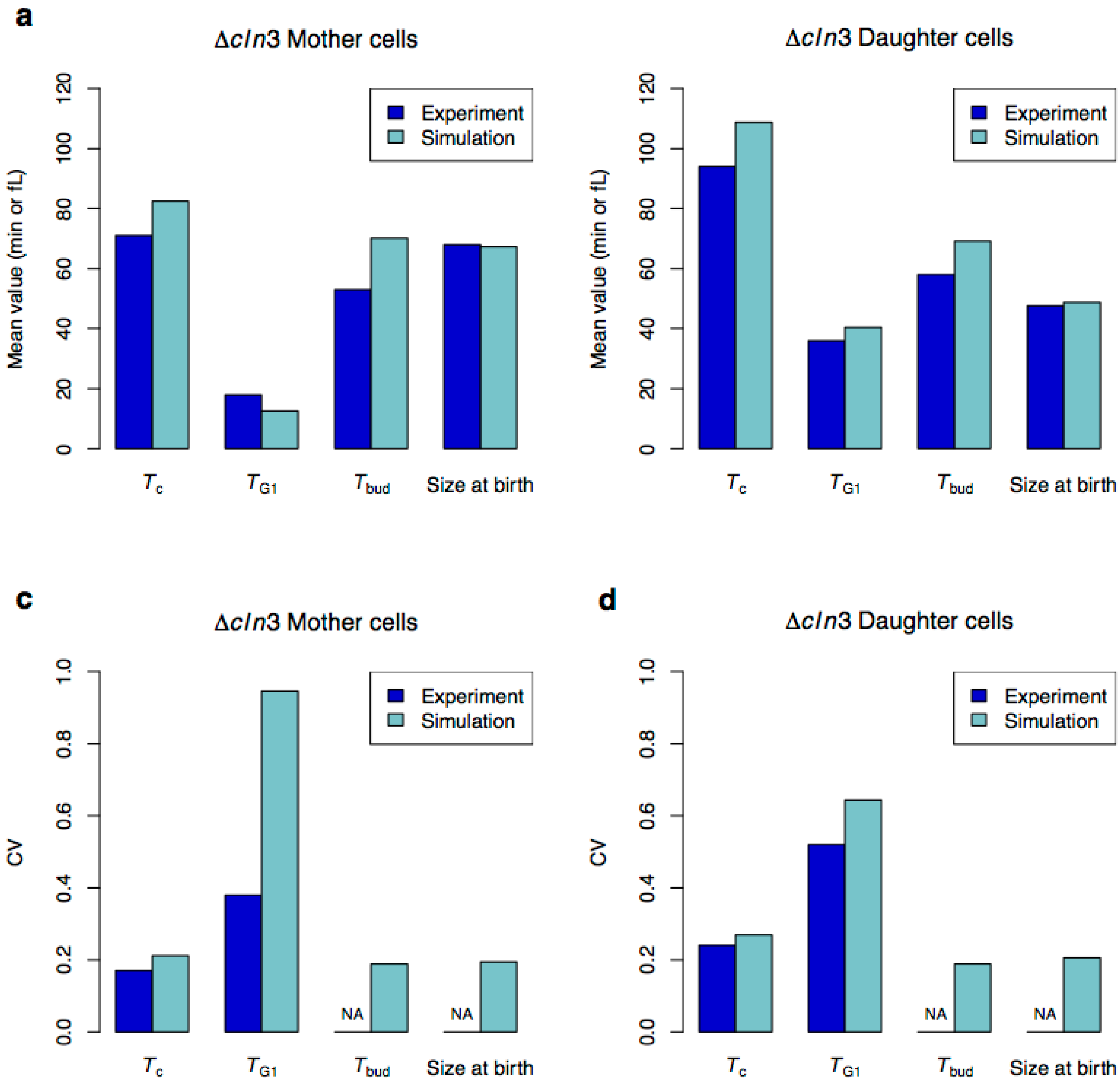

Supplementary Figure S7. Simulations of Δ*cln3* cells with S_{0_mean} = 0.8 (2.0×) of wild-type S_{0_mean}). (**a-d**) Mean and CV of cell-cycle properties computed from simulated populations of mother cells and daughter cells. Mean Size-at-birth of simulated Δ*cln3* mother cells is 1.68-fold larger than wild-type. Means and CVs of T_c, T_{G1}, and T_{bud} were reported by Charvin et al.[5] Size at birth, which is 1.7× wild-type, was observed in Dirick et al.[6]

Supplementary Table S1. Summary of Boolean models of eukaryotic cell cycle controls.

**Authors**<br>Year<br>Organism modeled	**Method**	**Results**
Li, Long, Lu, Ouyang & Tang[1]<br>2004<br>Budding yeast	Standard Boolean<br>11 variables<br>Synchronous updating	Robust sequence of cell-cycle transitions<br>$G_1/S \rightarrow G_2/M \rightarrow G_0$
Fauré, Naldi, Chaouiya & Thieffry[7]<br>2006<br>Mammalian cell	Standard Boolean<br>10 variables<br>Priority class updating	Alternative sequences from G_1/S to G_2/M to G_0<br>Mutant analyses provided on webpage
Fauré, Naldi, Lopez, Chaouiya, Ciliberto & Thieffry[8]<br>2009<br>Budding yeast	Standard Boolean<br>27 molecular variables<br>Synchronous updating<br>4 events + Mass	31-state cycle: $G_0/G_1 \rightarrow S/G_2 \rightarrow M \rightarrow G_0/G_1$<br>156 mutant strains simulated<br>(4 major discrepancies)
Davidich & Bornholdt[9]<br>2008<br>Fission yeast	Standard Boolean<br>10 variables<br>Synchronous updating	Robust sequence of cell-cycle transitions<br>$G_1/S \rightarrow G_2/M \rightarrow G_0$
Irons[10]<br>2009<br>Budding yeast	Standard Boolean<br>14 proteins + 4 events<br>Synchronous updating	19-state cycle<br>Correctly predicts the phenotypes of 13 mutant strains tested
Singhania, Sramkoski, Jacobberger & Tyson[11]<br>2011<br>Mammalian cell	Hybrid Boolean/ODE<br>deterministic/stochastic<br>6 Boolean + 4 continuous<br>Continuous time	Reproduces flow cytometry data on Cyclin A, Cyclin B and Cyclin E, and contact inhibition of a culture of human endothelial cells
Stoll, Viara, Barillot & Calzone[12]<br>2012<br>Mammalian cell	Stochastic Boolean<br>Stochastic asynch updating<br>Continuous time	Damped oscillations of Cyclin A, Cyclin B and Cyclin E
Davidich & Bornholdt[13]<br>2013<br>Fission yeast	Standard Boolean<br>12 variables<br>Synchronous updating	Correctly predicts the phenotypes of 32 mutant strains tested
Noël, Vakulenko & Radulescu[14]<br>2013<br>Mammalian cell	Hybrid Boolean/ODE<br>Generalize approach of Singhania et al.[11]	Apply to model of Csikasz-Nagy et al.[15]<br>Hybrid model compares favorably with nonlinear ODE model
Münzner, Klipp & Krantz[16]<br>2019<br>Budding yeast	Comprehensive Boolean<br>357 proteins, genes, mRNAs<br>Priority class updating	186-state cycle<br>Correctly predicts the phenotypes of 73% of 85 mutant strains tested
Howell, Klemm, Thorpe & Csikasz-Nagy[17]<br>2020<br>Budding yeast	Compartmental Boolean<br>37 variables<br>6 compartments<br>Asynch, random updating	Correctly predicts the phenotypes of 81% of 147 mutant strains tested
Laomettachit, Kraikivski & Tyson<br>(this study)<br>Budding yeast	Stochastic Boolean<br>7 discrete variables<br>+ Size (continuous)<br>Asynch, random updating	Reproduces statistical properties of cell cycle phase durations and cell size<br>91% of single-state perturbations return to normal sequence of cell cycle events

Standard Boolean model: discrete state variables (0 or 1), discrete time (0, 1, 2, …), deterministic updating (synchronous, asynchronous, or priorities given to certain variables).

Compartmental Boolean model: discrete state variables in each compartment (‘active’ or ‘inactive’, ‘present’ or ‘absent’), discrete time, deterministic updating.

Stochastic Boolean model: discrete state variables, random updating by Gillespie’s stochastic simulation algorithm.

Hybrid models: mixture of discrete and continuous variables, deterministic and stochastic updating.

Supplementary Table S2. All possible updates based on the logical functions, equations (7-13) in the main text.

**state(*t*)**	**state(*t*+1)**
0000000	1000000
0000001	1000001 0000000
0000010	0000110 0000011
0000011	0000111 0000001
0000100	0000110
0000101	1000101
0000110	0000111
0000111	0000101
0001000	0001100
0001001	0000001 0001101 0001000
0001010	0001110 0001011
0001011	0000011 0001111
0001100	0001110
0001101	0000101 0001111
0001110	0001111
0001111	0000111
0010000	0000000
0010001	0000001 0010000
0010010	0000010 0010110 0010000 0010011
0010011	0000011 0010111 0010001
0010100	0000100
0010101	0000101
0010110	0000110 0010100 0010111
0010111	0000111 0010101
0011000	0001000 0011100
0011001	0001001 0010001 0011101 0011000
0011010	0001010 0011110 0011000 0011011
0011011	0001011 0010011 0011111 0011001
0011100	0001100
0011101	0001101 0010101
0011110	0001110 0011100 0011111
0011111	0001111 0010111 0011101
0100000	1100000 0110000 0101000
0100001	1100001 0110001 0100000
0100010	0000010 0110010 0101010 0100110 0100011
0100011	0000011 0110011 0100111 0100001
0100100	0000100 0110100 0101100 0100110
0100101	1100101 0000101 0110101
0100110	0000110 0110110 0101110 0100111
0100111	0000111 0110111 0100101

**state(*t*)**	**state(*t*+1)**
0101000	0111000 0101100
0101001	0111001 0100001 0101101 0101000
0101010	0001010 0111010 0101110 0101011
0101011	0001011 0111011 0100011 0101111
0101100	0001100 0111100 0101110
0101101	0001101 0111101 0100101 0101111
0101110	0001110 0111110 0101111
0101111	0001111 0111111 0100111
0110000	0111000
0110001	0110000
0110010	0010010 0111010 0110110 0110000 0110011
0110011	0010011 0110111 0110001
0110100	0010100 0111100
0110101	0010101
0110110	0010110 0111110 0110100 0110111
0110111	0010111 0110101
0111000	0111100
0111001	0110001 0111101 0111000
0111010	0011010 0111110 0111000 0111011
0111011	0011011 0110011 0111111 0111001
0111100	0011100
0111101	0011101 0110101
0111110	0011110 0111100 0111111
0111111	0011111 0110111 0111101
1000000	1000000
1000001	1000000
1000010	0000010 1000110 1000000 1000011
1000011	0000011 1000111 1000001
1000100	0000100 1000000
1000101	1000001
1000110	0000110 1000100 1000111
1000111	0000111 1000101
1001000	0001000 1000000
1001001	0001001 1000001 1001000
1001010	0001010 1000010 1001110 1001000 1001011
1001011	0001011 1000011 1001111 1001001
1001100	0001100 1000100 1001000
1001101	0001101 1000101 1001001
1001110	0001110 1000110 1001100 1001111
1001111	0001111 1000111 1001101
1010000	0010000 1000000
1010001	0010001 1000001 1010000
1010010	0010010 1000010 1010110 1010000 1010011
1010011	0010011 1000011 1010111 1010001

**state(*t*)**	**state(*t*+1)**
1010100	0010100 1000100 1010000
1010101	0010101 1000101 1010001
1010110	0010110 1000110 1010100 1010111
1010111	0010111 1000111 1010101
1011000	0011000 1001000 1010000
1011001	0011001 1001001 1010001 1011000
1011010	0011010 1001010 1010010 1011110 1011000 1011011
1011011	0011011 1001011 1010011 1011111 1011001
1011100	0011100 1001100 1010100 1011000
1011101	0011101 1001101 1010101 1011001
1011110	0011110 1001110 1010110 1011100 1011111
1011111	0011111 1001111 1010111 1011101
1100000	1110000
1100001	1110001 1100000
1100010	0100010 1000010 1110010 1100110 1100000 1100011
1100011	0100011 1000011 1110011 1100111 1100001
1100100	0100100 1000100 1110100 1100000
1100101	1000101 1110101 1100001
1100110	0100110 1000110 1110110 1100100 1100111
1100111	0100111 1000111 1110111 1100101
1101000	0101000 1111000 1100000
1101001	0101001 1111001 1100001 1101000
1101010	0101010 1001010 1111010 1100010 1101110 1101000 1101011
1101011	0101011 1001011 1111011 1100011 1101111 1101001
1101100	0101100 1001100 1111100 1100100 1101000
1101101	0101101 1001101 1111101 1100101 1101001
1101110	0101110 1001110 1111110 1100110 1101100 1101111
1101111	0101111 1001111 1111111 1100111 1101101
1110000	0110000
1110001	0110001 1110000
1110010	0110010 1010010 1110110 1110000 1110011
1110011	0110011 1010011 1110111 1110001
1110100	0110100 1010100 1110000
1110101	0110101 1010101 1110001
1110110	0110110 1010110 1110100 1110111
1110111	0110111 1010111 1110101
1111000	0111000 1110000
1111001	0111001 1110001 1111000
1111010	0111010 1011010 1110010 1111110 1111000 1111011
1111011	0111011 1011011 1110011 1111111 1111001
1111100	0111100 1011100 1110100 1111000
1111101	0111101 1011101 1110101 1111001
1111110	0111110 1011110 1110110 1111100 1111111
1111111	0111111 1011111 1110111 1111101

Supplementary Table S3. Single-state perturbations. For each perturbation, the percentages are calculated from 5000 repeats. (When $Clb2_M$ is flipped on, $Clb2_G$ is also set to be on. When $Clb2_G$ is flipped off, $Clb2_M$ is also set to be off.)

State index	Pert'n index	Event (cell cycle phase)	State	Pert'n event	State after pert'n	% Reverting to G_1	% Resuming normal progression	% Exit without activating $Clb2_M$ or Cdc20	% Exit without budding
1	1	Newborn cell (G_1)	1000000	Cdh1 turns off	0000000	100.00	0.00	0.00	0.00
1	3	Newborn cell (G_1)	1000000	Cln2 turns on	1010000	100.00	0.00	0.00	0.00
1	4	Newborn cell (G_1)	1000000	Clb5 turns on	1001000	49.98	50.02	0.00	0.00
1	5	Newborn cell (G_1)	1000000	$Clb2_G$ turns on	1000100	50.40	0.00	0.00	49.60
1	6	Newborn cell (G_1)	1000000	$Clb2_M$ turns on	1000110	16.50	0.00	0.00	83.50
2	1	SBF turns on (bud emergence)	1100000	Cdh1 turns off	0100000	0.00	100.00	0.00	0.00
2	4	SBF turns on (bud emergence)	1100000	Clb5 turns on	1101000	0.00	100.00	0.00	0.00
2	5	SBF turns on (bud emergence)	1100000	$Clb2_G$ turns on	1100100	17.02	33.14	0.00	49.84
2	6	SBF turns on (bud emergence)	1100000	$Clb2_M$ turns on	1100110	8.18	8.04	0.00	83.78
2	7	SBF turns on (bud emergence)	1100000	Cdc20 turns on	1100001	0.00	100.00	0.00	0.00
3	2	Cln2 turns on	1110000	SBF turns off	1010000	100.00	0.00	0.00	0.00
3	4	Cln2 turns on	1110000	Clb5 turns on	1111000	0.00	100.00	0.00	0.00
3	5	Cln2 turns on	1110000	$Clb2_G$ turns on	1110100	15.82	84.18	0.00	0.00
3	6	Cln2 turns on	1110000	$Clb2_M$ turns on	1110110	8.18	91.82	0.00	0.00
3	7	Cln2 turns on	1110000	Cdc20 turns on	1110001	0.00	100.00	0.00	0.00
4	2	Cdh1 turns off	0110000	SBF turns off	0010000	100.00	0.00	0.00	0.00
4	3	Cdh1 turns off	0110000	Cln2 turns off	0100000	0.00	100.00	0.00	0.00
4	5	Cdh1 turns off	0110000	$Clb2_G$ turns on	0110100	0.00	100.00	0.00	0.00

State index	Pert'n index	Event (cell cycle phase)	State	Pert'n event	State after pert'n	% Reverting to G_1	% Resuming normal progression	% Exit without activating $Clb2_M$ or Cdc20	% Exit without budding
4	6	Cdh1 turns off	0110000	$Clb2_M$ turns on	0110110	0.00	100.00	0.00	0.00
4	7	Cdh1 turns off	0110000	Cdc20 turns on	0110001	0.00	100.00	0.00	0.00
5	1	Clb5 turns on (S phase)	0111000	Cdh1 turns on	1111000	0.00	100.00	0.00	0.00
5	2	Clb5 turns on (S phase)	0111000	SBF turns off	0011000	0.00	100.00	0.00	0.00
5	3	Clb5 turns on (S phase)	0111000	Cln2 turns off	0101000	0.00	100.00	0.00	0.00
5	6	Clb5 turns on (S phase)	0111000	$Clb2_M$ turns on	0111110	0.00	100.00	0.00	0.00
5	7	Clb5 turns on (S phase)	0111000	Cdc20 turns on	0111001	0.00	73.52	26.48	0.00
6	1	$Clb2_G$ turns on (prophase)	0111100	Cdh1 turns on	1111100	0.00	49.48	50.52	0.00
6	3	$Clb2_G$ turns on (prophase)	0111100	Cln2 turns off	0101100	0.00	100.00	0.00	0.00
6	4	$Clb2_G$ turns on (prophase)	0111100	Clb5 turns off	0110100	0.00	100.00	0.00	0.00
6	6	$Clb2_G$ turns on (prophase)	0111100	$Clb2_M$ turns on	0111110	0.00	100.00	0.00	0.00
6	7	$Clb2_G$ turns on (prophase)	0111100	Cdc20 turns on	0111101	0.00	23.04	76.96	0.00
7	1	SBF turns off	0011100	Cdh1 turns on	1011100	0.00	49.98	50.02	0.00
7	4	SBF turns off	0011100	Clb5 turns off	0010100	0.00	100.00	0.00	0.00
7	5	SBF turns off	0011100	$Clb2_G$ turns off	0011000	0.00	100.00	0.00	0.00
7	6	SBF turns off	0011100	$Clb2_M$ turns on	0011110	0.00	100.00	0.00	0.00
7	7	SBF turns off	0011100	Cdc20 turns on	0011101	0.00	46.48	53.52	0.00
8	1	Cln2 turns off	0001100	Cdh1 turns on	1001100	0.00	49.72	50.28	0.00
8	2	Cln2 turns off	0001100	SBF turns on	0101100	0.00	100.00	0.00	0.00
8	4	Cln2 turns off	0001100	Clb5 turns off	0000100	0.00	100.00	0.00	0.00
8	5	Cln2 turns off	0001100	$Clb2_G$ turns off	0001000	0.00	100.00	0.00	0.00
8	7	Cln2 turns off	0001100	Cdc20 turns on	0001101	0.00	50.82	49.18	0.00
9	1	$Clb2_M$ turns on (metaphase)	0001110	Cdh1 turns on	1001110	0.00	87.12	12.88	0.00

State index	Pert'n index	Event (cell cycle phase)	State	Pert'n event	State after pert'n	% Reverting to G_1	% Resuming normal progression	% Exit without activating $Clb2_M$ or Cdc20	% Exit without budding
9	2	$Clb2_M$ turns on (metaphase)	0001110	SBF turns on	0101110	0.00	100.00	0.00	0.00
9	3	$Clb2_M$ turns on (metaphase)	0001110	Cln2 turns on	0011110	0.00	100.00	0.00	0.00
9	4	$Clb2_M$ turns on (metaphase)	0001110	Clb5 turns off	0000110	0.00	100.00	0.00	0.00
9	5	$Clb2_M$ turns on (metaphase)	0001110	$Clb2_G$ turns off	0001000	0.00	100.00	0.00	0.00
10	1	Cdc20 turns on (anaphase)	0001111	Cdh1 turns on	1001111	0.00	100.00	0.00	0.00
10	2	Cdc20 turns on (anaphase)	0001111	SBF turns on	0101111	0.00	100.00	0.00	0.00
10	3	Cdc20 turns on (anaphase)	0001111	Cln2 turns on	0011111	0.00	100.00	0.00	0.00
10	5	Cdc20 turns on (anaphase)	0001111	$Clb2_G$ turns off	0001001	0.00	100.00	0.00	0.00
10	6	Cdc20 turns on (anaphase)	0001111	$Clb2_M$ turns off	0001101	0.00	100.00	0.00	0.00
11	1	Clb5 turns off	0000111	Cdh1 turns on	1000111	0.00	100.00	0.00	0.00
11	2	Clb5 turns off	0000111	SBF turns on	0100111	0.00	100.00	0.00	0.00
11	3	Clb5 turns off	0000111	Cln2 turns on	0010111	0.00	100.00	0.00	0.00
11	5	Clb5 turns off	0000111	$Clb2_G$ turns off	0000001	0.00	100.00	0.00	0.00
11	7	Clb5 turns off	0000111	Cdc20 turns off	0000110	0.00	100.00	0.00	0.00
12	2	$Clb2_M$ turns off (telophase)	0000101	SBF turns on	0100101	0.00	100.00	0.00	0.00
12	3	$Clb2_M$ turns off (telophase)	0000101	Cln2 turns on	0010101	0.00	100.00	0.00	0.00
12	4	$Clb2_M$ turns off (telophase)	0000101	Clb5 turns on	0001101	0.00	100.00	0.00	0.00
12	5	$Clb2_M$ turns off (telophase)	0000101	$Clb2_G$ turns off	0000001	0.00	100.00	0.00	0.00
12	7	$Clb2_M$ turns off (telophase)	0000101	Cdc20 turns off	0000100	0.00	100.00	0.00	0.00
13	2	Cdh1 turns on	1000101	SBF turns on	1100101	0.00	100.00	0.00	0.00
13	3	Cdh1 turns on	1000101	Cln2 turns on	1010101	0.00	100.00	0.00	0.00
13	4	Cdh1 turns on	1000101	Clb5 turns on	1001101	0.00	100.00	0.00	0.00
13	6	Cdh1 turns on	1000101	$Clb2_M$ turns on	1000111	0.00	100.00	0.00	0.00

State index	Pert'n index	Event (cell cycle phase)	State	Pert'n event	State after pert'n	% Reverting to G_1	% Resuming normal progression	% Exit without activating $Clb2_M$ or Cdc20	% Exit without budding
13	7	Cdh1 turns on	1000101	Cdc20 turns off	1000100	0.00	100.00	0.00	0.00
14	1	$Clb2_G$ turns off (Exit)	1000001	Cdh1 turns off	0000001	0.00	100.00	0.00	0.00
14	2	$Clb2_G$ turns off (Exit)	1000001	SBF turns on	1100001	0.00	100.00	0.00	0.00
14	3	$Clb2_G$ turns off (Exit)	1000001	Cln2 turns on	1010001	0.00	100.00	0.00	0.00
14	4	$Clb2_G$ turns off (Exit)	1000001	Clb5 turns on	1001001	0.00	100.00	0.00	0.00
14	6	$Clb2_G$ turns off (Exit)	1000001	$Clb2_M$ turns on	1000111	0.00	100.00	0.00	0.00

References

1 Li, F., Long, T., Lu, Y., Ouyang, Q. & Tang, C. The yeast cell-cycle network is robustly designed. *Proceedings of the National Academy of Sciences* **101**, 4781-4786, doi:https://doi.org/10.1073/pnas.0305937101 (2004).

2 Woldringh, C. L., Huls, P. G. & Vischer, N. O. Volume growth of daughter and parent cells during the cell cycle of *Saccharomyces cerevisiae* a/α as determined by image cytometry. *Journal of Bacteriology* **175**, 3174-3181, doi:https://doi.org/10.1128/jb.175.10.3174-3181.1993 (1993).

3 Chen, K. C. *et al.* Integrative analysis of cell cycle control in budding yeast. *Molecular Biology of the Cell* **15**, 3841-3862, doi:https://doi.org/10.1091/mbc.e03-11-0794 (2004).

4 Ball, D. A. *et al.* Stochastic exit from mitosis in budding yeast. *Cell Cycle* **10**, 999-1009, doi:https://doi.org/10.4161/cc.10.6.14966 (2011).

5 Charvin, G., Cross, F. R. & Siggia, E. D. Forced periodic expression of G_1 cyclins phase-locks the budding yeast cell cycle. *Proceedings of the National Academy of Sciences* **106**, 6632-6637, doi:https://doi.org/10.1073/pnas.0809227106 (2009).

6 Dirick, L., Böhm, T. & Nasmyth, K. Roles and regulation of Cln-Cdc28 kinases at the start of the cell cycle of *Saccharomyces cerevisiae*. *The EMBO Journal* **14**, 4803-4813, doi:https://doi.org/10.1002/j.1460-2075.1995.tb00162.x (1995).

7 Fauré, A., Naldi, A., Chaouiya, C. & Thieffry, D. Dynamical analysis of a generic Boolean model for the control of the mammalian cell cycle. *Bioinformatics* **22**, e124-e131, doi:https://doi.org/10.1093/bioinformatics/btl210 (2006).

8 Fauré, A. *et al.* Modular logical modelling of the budding yeast cell cycle. *Molecular BioSystems* **5**, 1787-1796, doi:https://doi.org/10.1039/B910101M (2009).

9 Davidich, M. I. & Bornholdt, S. Boolean network model predicts cell cycle sequence of fission yeast. *PLOS ONE* **3**, e1672, doi:https://doi.org/10.1371/journal.pone.0001672 (2008).

10 Irons, D. J. Logical analysis of the budding yeast cell cycle. *Journal of Theoretical Biology* **257**, 543-559, doi:https://doi.org/10.1016/j.jtbi.2008.12.028 (2009).

11 Singhania, R., Sramkoski, R. M., Jacobberger, J. W. & Tyson, J. J. A hybrid model of mammalian cell cycle regulation. *PLOS Computational Biology* **7**, e1001077, doi:https://doi.org/10.1371/journal.pcbi.1001077 (2011).

12 Stoll, G., Viara, E., Barillot, E. & Calzone, L. Continuous time boolean modeling for biological signaling: application of Gillespie algorithm. *BMC Systems Biology* **6**, 116, doi:https://doi.org/10.1186/1752-0509-6-116 (2012).

13 Davidich, M. I. & Bornholdt, S. Boolean network model predicts knockout mutant phenotypes of fission yeast. *PLOS ONE* **8**, e71786, doi:https://doi.org/10.1371/journal.pone.0071786 (2013).

14 Noël, V., Vakulenko, S. & Radulescu, O. A hybrid mammalian cell cycle model. *Electronic Proceedings in Theoretical Computer Science* **125**, 68-83, doi:https://doi.org/10.4204/EPTCS.125.5 (2013).

15 Csikász-Nagy, A., Battogtokh, D., Chen, K. C., Novák, B. & Tyson, J. J. Analysis of a generic model of eukaryotic cell-cycle regulation. *Biophysical Journal* **90**, 4361-4379, doi:https://doi.org/10.1529/biophysj.106.081240 (2006).

16 Münzner, U., Klipp, E. & Krantz, M. A comprehensive, mechanistically detailed, and executable model of the cell division cycle in *Saccharomyces cerevisiae*. *Nature Communications* **10**, 1308, doi:https://doi.org/10.1038/s41467-019-08903-w (2019).

17 Howell, R. S. M., Klemm, C., Thorpe, P. H. & Csikász-Nagy, A. Unifying the mechanism of mitotic exit control in a spatiotemporal logical model. *PLOS Biology* **18**, e3000917, doi:https://doi.org/10.1371/journal.pbio.3000917 (2020).
